# Thiol- and Disulfide-Based Stimulus-Responsive Soft Materials and Self-Assembling Systems

**DOI:** 10.3390/molecules26113332

**Published:** 2021-06-01

**Authors:** Danielle M. Beaupre, Richard G. Weiss

**Affiliations:** 1 Department of Chemistry, Georgetown University, Washington, DC 20057, USA; dmb301@georgetown.edu; 2 Institute for Soft Matter Synthesis and Metrology, Georgetown University, Washington, DC 20057, USA

**Keywords:** stimulus-responsive materials, self-healing, redox-responsive, drug delivery, thiol-disulfide exchange, disulfide-disulfide metathesis

## Abstract

Properties and applications of synthetic thiol- and disulfide-based materials, principally polymers, are reviewed. Emphasis is placed on soft and self-assembling materials in which interconversion of the thiol and disulfide groups initiates stimulus-responses and/or self-healing for biomedical and non-biomedical applications.

## 1. Introduction

### 1.1. Thiols and Disulfides in Materials Chemistry

Thiols (RSH) and disulfides (RSSR) are components of many proteins, biopolymers, and biomolecules. In nature, thiols and disulfides contribute to key biological functions related to cell signaling, protein conformation and folding processes, redox homeostasis, and biopolymer secondary structure development. Thiol groups and/or disulfide linkages can be incorporated into polymers to produce stimulus-responsive and/or self-healing materials. They owe their stimulus-responsiveness to covalent disulfide bonds, which can be broken and re-formed under mild conditions. The extent of reactivity, and thus the stimulus-responsive behavior depends on the nature of the groups flanking the disulfide bond, as well as interactions with stimuli such as light, heat, mechanical force, and changes in the pH or redox state [1]. The ease with which thiols can be converted to disulfides, and vice versa, renders thiol-containing polymers (thiomers) similarly dynamic.

This review will focus on the properties and applications of synthetic thiomers and disulfide-containing polymers, with an emphasis on soft and self-assembling materials. Common thiol-containing pendant groups, as well as the synthetic methodologies for their incorporation into polymers, have been discussed extensively in a 2019 review of biomedically relevant thiomers, which includes a discourse on thiomer design strategies [2]. A recent review by Bej and coworkers covered responsive aggregation of both small molecule and polymer amphiphiles containing disulfide bonds, with an emphasis on biomedical applications [3]. Additionally, thiol and disulfide-based self-healing materials were reviewed by Yang [4], Wang [5], Utrera-Barrios [6], and their coworkers. Here, we discuss polymers in which the thiol and disulfide groups are important to their function. In addition to the types of systems previously reviewed, many examples of disulfide-based, self-healing polymers, many of which are elastomers, will be mentioned. However, to the best of our knowledge, no review devoted specifically to self-healing disulfides has been published.

After a brief overview of reactions between (and among) thiols and disulfides, a variety of thiol- and disulfide-based stimulus-responsive systems will be reviewed. The examples will be classified by the structures of the materials and the stimuli employed. The processes include thiol-disulfide and disulfide-disulfide exchange reactions, and redox reactions, as well as multiple responses by materials that contain additional functionalities which trigger “cascade” reactions (such as decomposition). The connections between the applications and the structures of the materials and the nature of their reactions will be emphasized.

### 1.2. Some Important Reactions of Thiols and Disulfides

**Figure 1:**
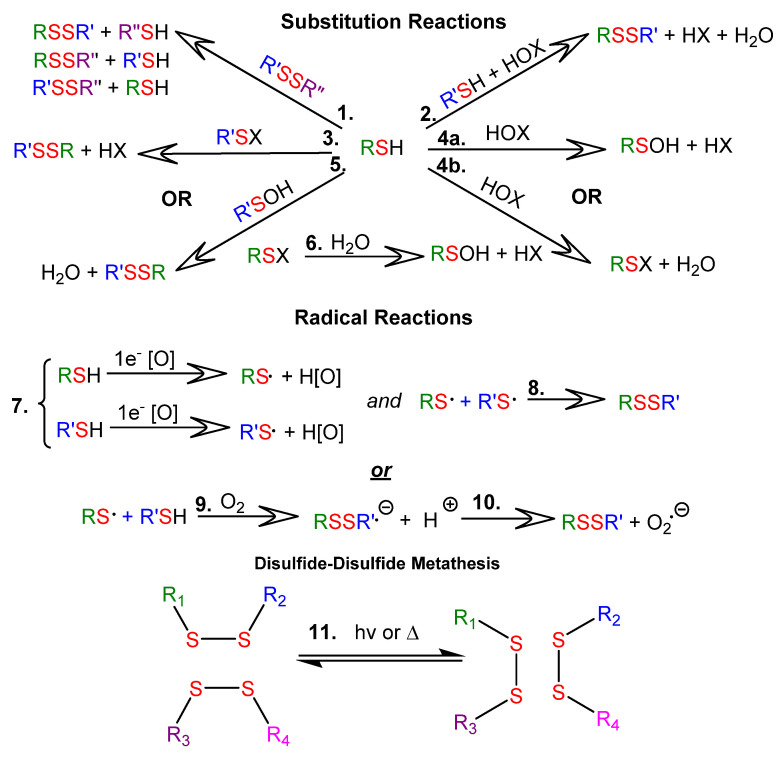
Reactions of thiols (RSH) and disulfides (RSSR): **1.1** S_N_2-mediated thiol-disulfide exchange; **1.2**–**1.6.** Thiol oxidation with two-electron oxidants, where RSX is a sulfenyl halide, RSOH is a sulfenic acid, HOX is a halohydrin, and HOx is the reduced oxidizing agent; **1.7**–**1.10** Thiol oxidation with one-electron oxidizing agents; **1.11** disulfide-disulfide metathesis.

Thiol-disulfide exchange reactions can occur between deprotonated thiols (thiolates) and disulfides. Thiol-disulfide exchange proceeds via substitution-type pathways, with the thiolate anion (RS^−^) functioning as the nucleophile (1 in Figure 1). In addition, thiols can be oxidized to disulfides (2 through 10 in Figure 1), and disulfides can be reduced to thiols. Although the products of oxidation or reduction reactions are often the same as those of thiol-disulfide exchange reactions, the pathways are mechanistically different (as shown in Figure 1). There are well-documented multi-step examples of both one electron (7 through 10 in Figure 1) and two electron (2 through 6 in Figure 1) oxidation pathways within cells [7]. Thiyl radicals (RS⋅) can be generated from thiols or disulfides using a variety of reagents or stimuli. Thiyl radicals play a crucial role in biological systems and are important reagents in organic synthesis. Thiyl radicals are intermediates in disulfide-disulfide metathesis reactions, also known as disulfide-disulfide exchange reactions, which proceed via a radical pathway (11 in Figure 1). Because disulfide linkages are often the weakest covalent bonds in an organic molecule, they provide an avenue to tune the properties of a material under mild reaction or stimulus conditions. Please note that the product distributions of both thiol-disulfide and disulfide-disulfide exchange depend upon the ratios of reactants and the nature of the R groups, which determine product stabilities.

### 1.3. Biological Relavance of Thiols and Disulfides

**Figure 2:**
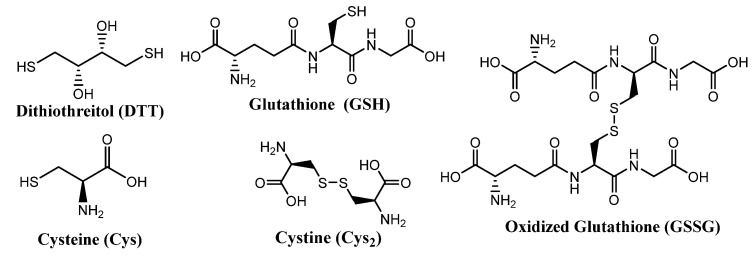
Structures of some biologically important thiols and disulfides.

Some molecules of biological relevance that exploit the disulfide bond are shown in Figure 2. For example, processes involving cystine (Cys_2_) and cysteine (Cys) interconversions have structural and functional consequences on the macroscopic and microscopic scale. On a macroscopic level, disulfide bonds in Cys_2_ stabilize the secondary structure of keratin-based materials, such as hair, wool, and nails. The cleavage of disulfide bonds in keratin results in a dramatic decrease of strength and elasticity of these materials. In that regard, the redox states and pH of intracellular and extracellular environments control the reversible oxidation of Cys to Cys_2_ in polypeptides. As such, disulfide bonds are keys to the protein folding process, and thus strongly dictate protein conformation.

### 1.4. Thiols and Disulfides in Materials Science

Nature has shown that thiol-disulfide exchange and redox reactions can be used to interconvert thiols and disulfides reversibly in response to changes in pH or redox state. The dynamic nature of the disulfide bond has been extended to materials science to produce a wide range of stimulus-responsive materials. The reviews of Seidi [8] and Zhang [9] include recent examples of controlled drug release, with an emphasis on stimulus-responsive and redox-triggered drug release. These reviews include several examples of thiol- and disulfide-based materials.

Thiol-disulfide exchange is often used to bind thiomers to disulfide-containing substrates, and to trigger the release of disulfide bound “cargo,” such as drugs or dyes, from a polymer upon exposure to high concentrations of thiols and/or changes in pH. Thiol oxidation and disulfide reduction are commonly used to form and break covalent disulfide crosslinks in polymeric networks, respectively. The scission and reformation of disulfide crosslinks in situ via reduction and oxidation, respectively, is often reported as a means for reversible sol-gel transitions. Commonly, dramatic rheological changes are observed over several reversible sol-gel redox cycles. Self-healing behavior has been widely reported for a variety of disulfide-containing polymers, due to disulfide-disulfide shuffling reactions, which occur after breakage.

### 1.5. Applications of Thiomers and Disulfide-Containing Polymers

**Figure 3:**
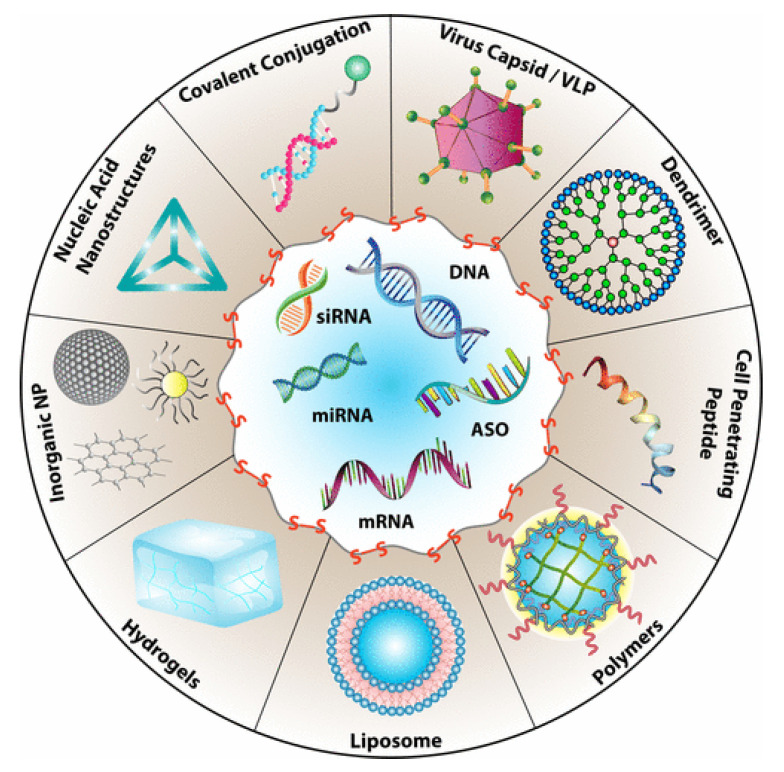
Types of drug delivery systems that rely on the presence of disulfide bonds for the targeted delivery of nucleic acids. Reprinted with permission from [10]. Copyright (2021) American Chemical Society.

Drug delivery is among the most common biomedical applications of thiomers and disulfide-containing polymers. The prototypical drug delivery system releases a conjugated drug molecule after being exposed to a cellular trigger. For example, solid tumors have higher levels of GSH and lower pH levels than healthy tissues. Chemotherapy employing a drug delivery system can be tuned to release the drug when exposed to the unique chemical environment of a cancer cell; more targeted drug delivery results in fewer side effects [3]. Figure 3 demonstrates the wide variety of drug delivery systems that rely on disulfide bonds for controlled delivery of nucleic acid therapeutics [10].

The reviews of Dutta [10], Wang [11], and Saitoa [12] focus on disulfide-based drug delivery systems. Another notable application is in the field of biomedicine, which merges biological and physiological principles within clinical science. Biomedicine often deals with mucoadhesive materials, which contain thiols and/or disulfides that penetrate mucosal membranes by binding with free thiol side chains from Cys residues [2].

The applications of thiomers and disulfide-containing polymers extend beyond biomedicine. A wide range of dynamic materials, such as self-healing elastomers [4,13], shape memory polymers [14] and stimulus-responsive polymers [9,15] have been designed as coatings [16], adhesive materials [17], and sensors [2,18]. Incorporation of thiols and/or disulfides into polymers that contain other stimulus-responsive moieties is an emerging technique that has been used to fabricate multi stimulus-responsive materials that may enhance specificity for both biomedical [15] and non-biomedical applications [6,14,18] For example, drug delivery systems incorporating multi-stimulus-responsive materials can increase the amount of drug reaching the targeted cells [19,20]. Some multi stimulus-responsive materials have been designed to release a drug only after the application of two or more stimuli associated with the target environment. Other multi stimulus-responsive drug delivery systems incorporate functional groups designed to enhance targeting specific types of cells.

## 2. Factors Influencing the Reactivity of Thiols and Disulfides

### 2.1. Factors Influencing the Bond Strengths of Thiols and Disulfides

The strengths of both the RS-H and RS-SR bonds are highly dependent upon the nature of the attached R groups. Thus, changing the R group provides a means to tune the reactivity and, therefore, the stimulus-responsiveness of thiol- and disulfide-containing materials. Alkanethiols and thiophenols have homolytic S-H bond dissociation energies of ca. 87 and 79 kcal/mol, respectively [21]. Disulfides generally have lower homolytic S-S bond dissociation energies, ranging from 50-65 kcal/mol [21]. Aryl thiols, such as thiophenol, generally have the lowest homolytic S-H bond dissociation energies, especially when an electron donating aryl substituent is present at the *para* position [22]. The relatively low homolytic bond dissociation energies of thiols and disulfides facilitate important radical reactions, and thiols are very popular initiators for free radical polymerization reactions [22].

### 2.2. Reactivity of Thiols and Disulfides in Thiol-Disulfide Exchange Reactions

Thiol pKa values span a large range, from 3 to 11, with values contingent upon the nature of the R group attached to the sulfur atom [23]. Often, pKa values can be used to predict whether a reaction will favor the reactants or products in a thiol-disulfide exchange (1 in Figure 1). The thiolate anion RS^−^ reacts about 10^10^ times faster than the corresponding thiol in a thiol-disulfide exchange reaction. However, because thiol-disulfide exchange is known to be a kinetically driven process, equilibrium parameters are sometimes not good predictors [7].

**Figure 4:**
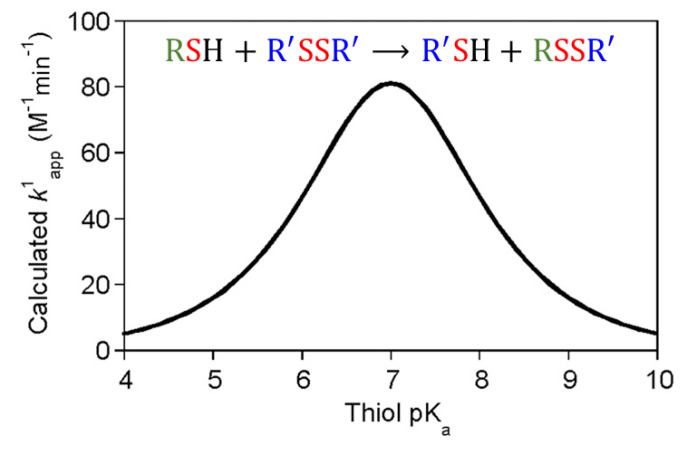
Calculated apparent rate constant for thiol-disulfide exchange in a solution at pH 7 as a function of reactant thiol pKa Reprinted with permission from [7]. Copyright Mary Ann Liebert, Inc.

When the pKa of the thiol is ~7, the thiol-disulfide exchange rate constant is at its maximum value (Figure 4; [7]). The rate constant decreases as thiol pKa decreases below 7, or increases above 7, due to a decrease in thiolate nucleophilicity and in the fraction of thiolate in solution. In addition to pKa values, a comparison of the redox potentials of the thiol reactant and product is generally a successful method to predict whether a reaction is favored [7,24].

**Figure 5:**
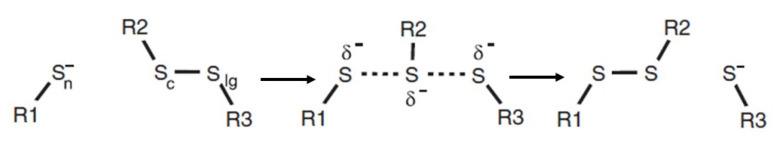
The orientation of a thiol and disulfide during thiol-disulfide exchange. S_n_ is the nucleophilic sulfur of the thiolate reactant; S_c_ and S_lg_ are the central and leaving group sulfur atoms of the disulfide reactant Adapted with permission from [23]. Copyright (2013) Elsevier B.V.

However, considering only the nature of the R groups of the thiol reactant and product tends to be a pragmatic (rather than critically planned) approach when the reactants and/or products are polymers. The nature of monomers and co-monomers along the polymer backbone must also be considered. All else being equal, a thiomer comprised of a flexible, low glass transition temperature (T_g_) polymer backbone will be more reactive than one with a strong, rigid polymer backbone because the thiol and disulfide groups in the former are more likely to adopt the required transition state geometry (Figure 5) when they are nearer. For similar reasons, similar polymers with greater degrees of thiolation are more reactive to a disulfide than those with lower degrees of thiolation.

**Figure 6:**
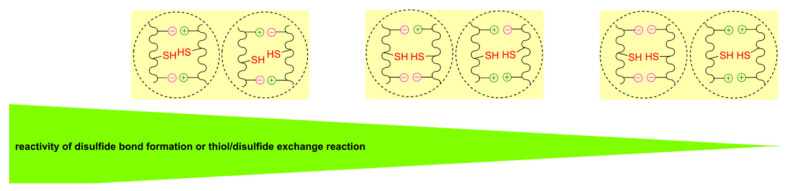
Cartoon showing the influence of electrostatic effects of R groups flanking thiols on their relative rates of thiol-disulfide exchange reactions. Adapted with permission from [2]. Copyright (2019) Elsevier B.V.

Disulfide products are stabilized by electron donating groups, the presence of negatively charged groups, and hydrogen bonding in the overall structure [2]. The placement and type of electrostatic charges on the backbone and the groups surrounding the thiol or disulfide influence the reactivity in the exchange reaction. Reactivity is inhibited if the thiol or disulfide moieties are flanked by groups with the same charge type. Similarly, if both the thiol and the disulfide have the same charge, the rate of exchange will be impeded by electrostatic repulsion (Figure 6; [2]).

Some thiol reactants can yield cyclic disulfides in thiol-disulfide exchange reactions. Likewise, some disulfide reactants can yield thiols that tautomerize into forms such as thiones. In either case, the reverse reaction is substantially slowed and effectively blocked, resulting in a near quantitative thiol-disulfide exchange [23,24]. Thiols and disulfides, such as dithiothreitol (DTT) and 4,4′dithiodipyridine, are therefore favored reactants for the reduction of disulfides and the oxidation of thiols, respectively [23]. Although these reagents participate mechanistically in thiol-disulfide exchange reactions, the products are formed in nearly quantitative yields; thus, the terms “oxidation” and “reduction” are far more common in the literature. Conversely, the term “thiol-disulfide exchange” is more commonly used to describe reactions that are more dynamic and not quantitative.

### 2.3. Factors Influencing the Redox Chemistry of Thiols and Disulfides

#### 2.3.1. Reduction of Disulfides with Thiols by Exchange Reactions

Reactions of disulfides with redox active thiols, such as DTT, are frequently described in the literature as being reductions, but mechanistically, they are often thiol-disulfide exchange reactions [7]. Generally, the term “reduction” is applied to thiol-disulfide exchange reactions that are occur in nearly quantitative yields. This condition can be achieved by employing excess thiol reagent and using specific thiols that have higher reduction potentials or that form cyclic rings (such as mercaptoethanol (ME) or DTT; Figure 2).

DTT and GSH are the most popular reagents for the in situ reduction of disulfide gels to thiol sols In soft matter, and many studies have shown that thiol sols can be reversibly oxidized to disulfide gels, then reduced with DTT or GSH to regenerate the thiol sol. This process can often be repeated several times with little loss of disulfide gel strength in some cases [25,26]. As such, it is ideal for applications related to fast and reversible in situ gelation. However, in depth analysis of the gels and sols is not possible if the by-products are not removed between the gel-sol transformation steps.

#### 2.3.2. Direct Reduction of Thiols with Non-Thiol Reducing Agents

Facile, direct reduction of disulfides to thiols can be achieved with several non-thiol reducing agents, such as borohydrides, zinc and dilute acid, nascent hydrogen, and phosphines [24,27]. The direct reduction of disulfides with non-thiol reductants does not require the removal of disulfide by-products or residual thiol reductants in the reaction mixture. As indicated in the previous section, by-products that cannot be easily removed can often complicate analysis. It is also possible to reduce one disulfide to one equivalent of the corresponding thiol and one equivalent of an S-substituted derivative using sulfite or cyanide. Although the atom efficiency of such reactions is low, they are used often for analytical purposes [24].

### 2.4. Oxidation of Thiols to Disulfides

The oxidation of thiols can produce both symmetrical and unsymmetrical disulfides, the latter of which are sought for a variety of applications [28,29]. The formation of unsymmetrical disulfides has been extensively used to determine important members of dynamic combinatorial libraries comprised of thiol and disulfide mixtures. The knowledge gained from studying dynamic combinatorial libraries of thiols and disulfides can be used to promote specific reaction products, and interesting results have been reported for many unsymmetrical systems [1,30].

The groups attached to a thiol determine how easily it can be oxidized to a disulfide. The ease of oxidation increases from tertiary to primary R groups attached to sulfur, and aryl thiols are easier to oxidize than aliphatic ones. Aryl thiols can often be oxidized by molecular oxygen, even in air [28]. However, over-oxidation of thiols to sulfonic acids, sulfinc acids, etc. can occur with some oxidants. Iodine and disulfide-based reagents have been found less likely to over-oxidize thiols beyond the disulfide form. The oxidation of tertiary thiols generally requires very harsh conditions that are not tolerated by most functional groups. Conversely, the aerobic oxidation of aryl and primary thiols to the corresponding disulfides can often be achieved in high yields in the presence of a silica-supported iron, manganese, or cobalt salt catalysts [28]. Unlike air, diethyl azodicarboxylate (DEAD) and diisopropyl azodicarboxylate (DIAD) can be used to selectively form unsymmetrical disulfides, RSSR’ from two thiols, RSH and R’SH [28,29].

There are many reports of materials based on aryl disulfides that exhibit exceptional self-healing abilities [4,13]. Overall, aryl and primary disulfides, as well as a wide variety of non-aliphatic disulfides, are favored in self-healing applications because they are sufficiently reactive to undergo facile oxidation or thiol-disulfide exchange reactions. The mild conditions needed for aryl disulfide S-S bond scission generally tolerate most of the common polymer backbone functional groups. Thus, aryl disulfides are well suited for incorporation into multi stimulus-responsive materials, which often contain other functional groups. Many other materials incorporating aryl disulfides are more sensitive to applied stimuli than are aliphatic disulfides [13]. For that reason, employing aryl disulfides facilitates finding conditions appropriate for provoking material response. A potential problem of incorporating aryl disulfide groups into materials is the possibility of decomposition via hydrolysis at higher pH values [24]. As pointed out by Leichner [2], thiol- or disulfide-containing polymers behave optimally when two competing factors—strong responses to applied stimuli in the target environment, and stability before entering the target environment—are balanced. If thiomers or disulfides are too reactive, they may prematurely react with non-target stimuli, or decompose before reaching target sites in the body.

### 2.5. Radical Reactions and Radical Coupling of Thiols and Disulfides

Disulfide-disulfide metathesis (11 in Figure 1) is the primary reaction responsible for the self-healing processes of many disulfide-based polymers and elastomers [4]. Although many such polymers have been reported to be self-healing, the mechanistic process for it is not well understood.

Disulfide-disulfide metathesis is also an important side reaction in many disulfide-containing polymers [31]. When materials containing disulfide bonds are mechanically fractured, thiyl radicals are generated [4,32]. Self-healing of fractured disulfide-containing polymers requires thiyl radical chain ends to approach one another, after which radical recombination and self-healing become possible [4]. Self-healing is favored within flexible polymers with low T_g_ values, and recent research has considered various methods to incorporate disulfides into high T_g_ polymers to produce stronger materials that can adequately self-heal [13]. Disulfide-containing polymers that are slow to self-heal under ambient conditions can be exposed to ultraviolet (UV) light or heat to accelerate the rate and efficiency of self-healing.

## 3. Reaction Types and Applications

Thiols and disulfides have been incorporated into a wide variety of polymer backbones to impart stimulus-responsiveness. In many cases, an applied stimulus prompts either a thiol-disulfide exchange reaction or a redox reaction. The review of Leichner contains examples of common polymer backbones and common thiol-containing pendant groups which can be appended to them to generate thiomers [2]. The wide array of polymer backbones reported in the literature reflects why thiomers and disulfide-containing polymers are so ubiquitous in materials science: thiols and disulfides can be incorporated into many polymers with relative ease by a variety of techniques [1]. Additionally, the reactivity of the thiol and disulfide groups can be tailored easily by altering the connectivity (changing R groups). Thiols and disulfides are ubiquitous components of living systems, which have evolved sophisticated and specific reactivity pathways for in vivo stimulus-responsiveness. Thiomers and disulfide-containing polymers are ideal candidates for applications where biological stimuli are desired to trigger a response [2]. The following sections are divided to show key methods in which stimuli have been used to prompt thiol-disulfide exchange, disulfide-disulfide exchange, and/or redox reactions for specific applications.

### 3.1. Thiol-Disulfide Exchange-Based and Redox Reactions

#### 3.1.1. Natural Keratin Protection and Regenerated Keratin Enhancement

There is much interest in finding methods by which keratin waste products can be processed into high quality materials. Keratin is a ubiquitous, crosslinked biopolymer protein that forms a variety of materials, including animal horns, feathers, nails, and hair [33]. The animal butchery and textile industries produce large volumes of keratinous waste materials that usually are not repurposed due to a large decrease in the mechanical properties suffered during processing [34]. That is unfortunate because keratin is one of the strongest known natural materials. The currently employed conditions are harsh, in that they rupture disulfide bonds and destroy secondary structures [35,36].

A large part of the strength of keratin is a consequence of the large number of disulfide bonds between Cys groups of its amino acid residues [37]. In combination with strong hydrogen bonding, the disulfide bonds in keratin enable the formation of very strong and stable secondary structures, such as fibers.

**Figure 7:**
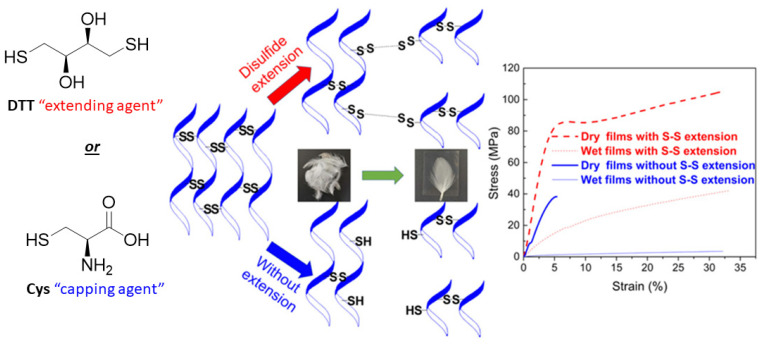
Structures of disulfide extenders (in red, DTT) and capping agents (in blue, Cys) that were added to processed keratin to restore disulfide bonds. Adapted with permission from [38]. Copyright (2020) American Chemical Society.

Mi et al. demonstrated that the secondary structures of waste keratinous materials can be restored after processing by crosslinking it with a dithiol, dithiothreitol (DTT), which functions as a “disulfide chain extender” [38]. In their work, the disulfide bonds of keratin were reduced, after which either a “disulfide capping agent” (Cys) or a “disulfide chain extender” (DTT) was added to regenerate disulfide bonds (Figure 7). Both wet and dry thin films fabricated from the disulfide chain extended keratin exhibited superior mechanical properties, relative to the capped keratin films.

The capping agents and disulfide extending groups were incorporated by reacting the free thiol (Cys) groups of processed keratin with individual Cys molecules or DTT. Unlike the capped keratin, the disulfide extended keratin can form extended networks because one molecule of DTT can react with itself and/or the Cys groups in keratin, forming DTT-based disulfide bridge crosslinks of variable lengths. Mi et al. hypothesize that the chain extended, crosslinked keratin network is dynamic, and that disulfide-disulfide metathesis facilitates crosslink reorganization during stretching (thus making the elasticity and tensile strength of the disulfide extended keratin film greater than the capped keratin film). The different bridge lengths are hypothesized to be the main reason for the dynamic nature of the disulfide extended network.

**Figure 8:**
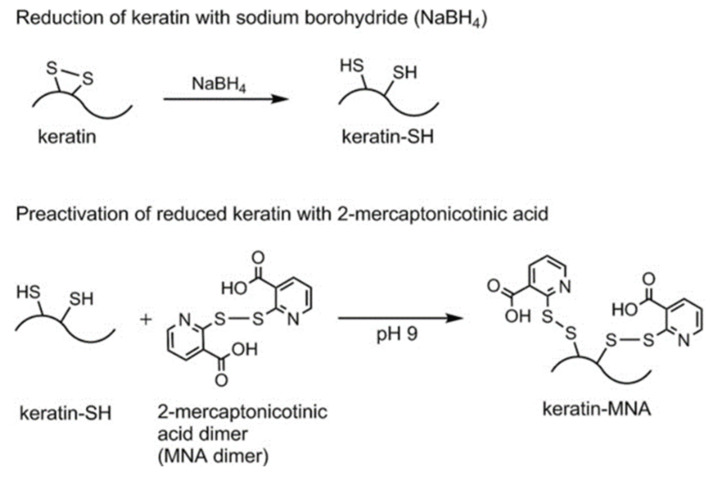
Reduction of keratin, to generate keratin-SH, and subsequent reaction with oxidized MMA to generate keratin-MMA via a thiol-disulfide exchange reaction Reprinted with permission from [39]. Copyright (2019) Elsevier B. V.

Other groups have adopted different approaches to investigate how to protect keratin by adding a thiomer capping agent. Thiol groups are an ideal target site for attachment of groups that can protect or restore hair, because hair contains between 7–20% Cys [37], most of which is present in the oxidized Cys_2_ form [39]. Leichner and coworkers [39] reduced keratin from human hair, generating a form with free Cys thiol groups (keratin-SH). They further derivatized keratin-SH with oxidized 2-mercaptonicotinic acid (MNA), generating a keratin-MNA derivative (Figure 8).

Natural and bleached hair strands were soaked in an aqueous solution at pH 8 containing 1% of either keratin, keratin-SH, or keratin-MMA, to determine the binding ability of the keratins to hair. Keratin-MMA had the highest binding affinity to both natural and bleached hair fibers, relative to underivatized keratin and keratin-SH. The increase in the binding ability of keratin-MMA was especially pronounced for bleached hair strands, which were able to bind approximately three times more keratin-MMA than underivatized keratin, regardless of whether the strands were exposed to one or five treatments noted above. Fernandes [40], Roddick-Lanzilotta [41], and Barba [42] also investigated alternative hair treatments designed to avoid directly breaking and then reforming disulfide bonds in keratin.

Fundamentally, both the protection and regeneration of keratinous materials relies on an initial reduction followed by a binding step that establishes new disulfide bond crosslinks between the Cys groups of keratin and thiol groups of the added material.

#### 3.1.2. Mucoadhesion

**Figure 9:**
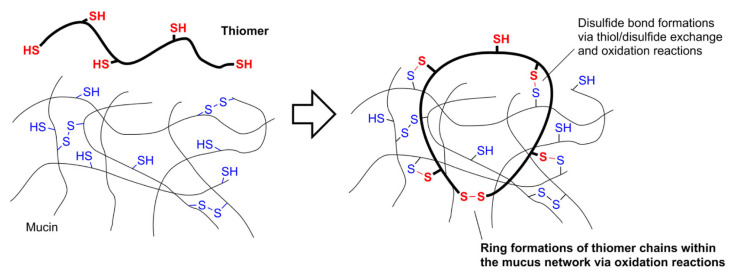
Cartoon showing key reactions and processes occurring during mucoadhesion. Reprinted with permission from [2]. Copyright (2019) Elsevier B. V.

Thiomers and disulfide-containing polymers have garnered interest as potential mucoadhesive drug delivery carriers. The use of thiomers and disulfide-containing polymers, also-called “S-protected” polymers, as mucoadhesives has been the subject of several reviews in the last decade [43,44,45,46], and drugs formulated with mucoadhesive thiomers have been successfully developed and marketed. In 2014, Lacrimera^®^ eyedrops, formulated with thiolated chitosan, were introduced to European markets [47]. Mucosal membrane surfaces are coated with cysteine-rich mucin glycoproteins at a variety of key sites in the body, including the mouth and nose. Permeation enhancing materials can be added to increase drug permeation through membranes [48], and the size of the permeation enhancer determines whether it will be absorbed through the mucosa and taken into the bloodstream along with the drug—which is the case for small molecules—or whether it will permeate the mucosal layer and remain bound to it during, and after, drug absorption—which is the case with polymers. Macromolecular mucoadhesive materials are less invasive because they do not enter the blood stream. Mucoadhesion was achieved through a variety of non-covalent interactions, such as van der Waals forces, hydrogen bonding, and hydrophobic interactions [49]. Thiomers and disulfide-containing mucoadhesive polymers; however, covalently bind to mucosa through the formation of new disulfide bonds between the Cys and/or Cys_2_ of mucin and disulfides and/or thiols on the polymer. Thiol-disulfide exchange reactions between the polymer and mucosa prompt in situ gelation that binds the polymer to the membrane [50]. Like the systems described in Section 3.1.1, the key factor that allows thiomers to function in this application is binding of a thiomer to a protein-rich substrate via a disulfide bond formed in a thiol-disulfide exchange reaction (Figure 9). The newly formed disulfide bonds can also participate in disulfide-disulfide metathesis reactions, affecting the quality of membrane adhesion through the formation of various inter- and intra-molecular disulfide crosslinks. Oxidation of thiols by GSH, which is present in mucosal membrane cells, is also important to the binding of thiomers to mucosa. The reactivity of thiomers and S-protected thiomers changes as a function of the penetration depth into the mucosal membrane, as a response to decreasing pH [51].

Thiolation of polymer backbones derived from naturally occurring biopolymers, like hyaluronic acid [52], chitosan [53,54], alginate [55], gellan gum [56,57], starch [58], glycogen [59], cellulose [60,61], arabinoxylan [62], xanthan gum [63,64], and cyclodextrins [65,66,67] have been studied as mucoadhesive materials. The mucoadhesive properties of thiolated synthetic polymers have been studied, also. Synthetic polymer backbones for this purpose include: poly(acrylic acid) [68,69], poly(methacrylate) [70], poly(acrylamide) [71], silicone oil [72,73], poly(ethylene glycol) (PEG) [74,75], poly(aspartamide) [76], and poly(allylamine) [77]. Thiolation of both hydrophobic and hydrophilic polymers generally increases mucoadhesivity [2].

**Figure 10:**
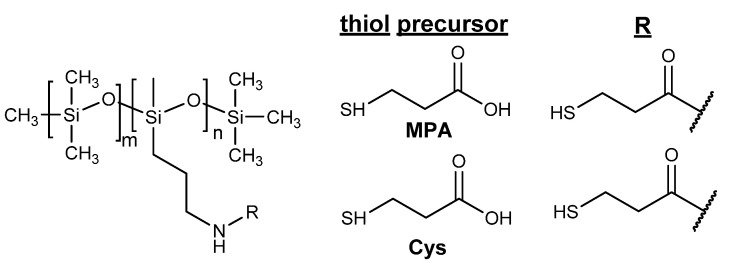
Thiolated silicone oils bearing 3-mercaptopropionic acid (MPA) or cysteine (Cys) pendant groups synthesized by Partenhauser et al. [72].

Partenhauser and coworkers attached thiol pendant groups to poly(dimethylsiloxane)-co-(3-aminopropyl)methylsiloxane by forming amide bonds between the carboxylic acid group of a thiol “ligand” precursors—either 3-mercaptopropionic acid (MPA) or Cys—and the primary amine groups of the poly(siloxane) (Figure 10; [72]).

**Figure 11:**
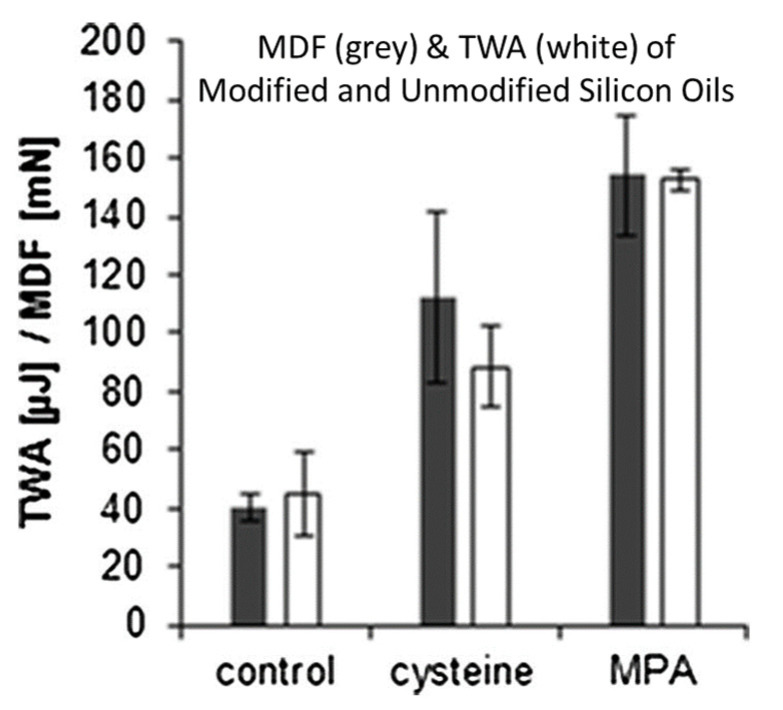
Maximum detachment force (MDF, gray bars) and total work of adhesion (TWA) were calculated as the area under the force versus displacement curves. Reprinted with permission [72]. Copyright (2015) Elsevier.

Mucoadhesion between a silicone oil and excised porcine mucosa was measured for silicone oil-MMA and silicone oil-Cys, as well as a non-thiolated silicone oil control (Figure 11). The maximum detachment force required to separate the mucosa from the silicone oil, as well as the total work of adhesion values demonstrate that the thiolated silicone oils bond more strongly to the mucosa than does the non-thiolated control.

The residence time of the silicone oils on porcine mucosa was measured after submerging the samples in a phosphate buffer solution (pH 6.8). The non-thiolated silicone oil was completely washed away after about 2 h in the solution, whereas 40–60% of the thiolated silicone oil remained on the surface after 8 h for silicone oil-MPA and silicone oil-Cys.

#### 3.1.3. Organic/Inorganic Hybrid Materials and Thiolated Organosilica Nanoparticles

Thiol- and disulfide-containing organosilica materials have been used for other applications, moreover mucoadhesion. For example, organosilica nanoparticles (ONPs) have been investigated as potential nanotherapeutics for anti-cancer drug delivery. ONP-based drug delivery systems tend to be physically stronger than other well-studied drug delivery system platforms based on liposomes or micelles [78]. The use of nanosized drug delivery systems for anticancer drug delivery offers advantages because cancer cells tend to accumulate selectively nanosized materials and retain them to a greater extent than non-nanosized materials (possibly due to enhanced permeability and retention [79]). Additionally, the unique behavior of nanosized materials in magnetic fields, and in response to light, opens possibilities for manipulating nanomedicines in the body. Disulfide-containing materials are an attractive choice for improving controlled release of anticancer drugs in response to elevated levels of the reducing agent, glutathione (GSH; Figure 2), in cancer cells. A drug delivery system should be sufficiently strong so that disulfide bonds linking the drug to the substrate do not break prematurely in cells that have normal cellular GSH levels. In an ideal system, disulfide bond scission will prompt anticancer drug release from the drug delivery system substrate only within the cancer cell. The concentration of GSH in cancer cells ranges between 2–10 mM, which is 100-1,000 times greater than in non-cancer cells [80].

**Figure 12:**
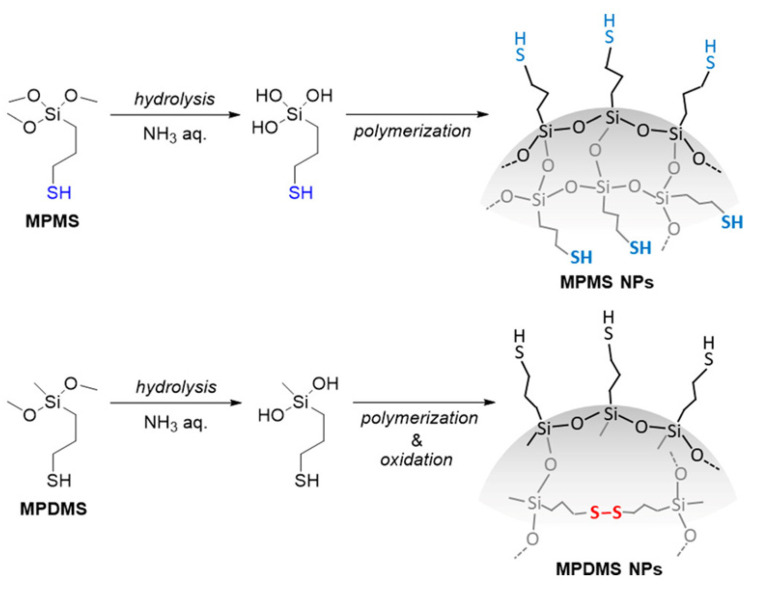
Structures of organosilica nanoparticles (ONPs) derived from (3-mercaptopropyl)trimethoxysilane (MPMS) and (3-mercaptopropyl)methyldimethoxysilane (MPDMS) precursors. Reprinted with permission from [81]. Copyright (2018) American Chemical Society.

Mekaru et al. used soft X-ray photoelectron spectroscopy (XPS) to study the biodegradability of two disulfide-containing ONPs formed from the hydrolysis of (3-mercaptopropyl)trimethoxysilane (MPMS) or (3-mercaptopropyl)methyldimethoxysilane (MPDMS) (Figure 12; [81]).

The sol-gel synthesis of MPMS ONPs via base hydrolysis resulted in ONPs with a greater degree of siloxane crosslinking than the MPDMS ONPs, because MPMS can form up to three siloxane bonds per monomer, whereas MPDMS can only form one siloxane bond per monomer. Consequently, the MPMS ONPs were expected to be more extensively crosslinked, more compact, and more rigid than MPDMS, but to have fewer disulfide crosslinks due to greater conformational restriction in the network. To demonstrate that the ONPs would decompose through disulfide bond scission, Meraku et al. incubated the ONPs in 10 mM GSH and monitored their degradation over 7 days using Raman spectroscopy and soft X-ray photoelectron spectroscopy (XPS), both of which are able to differentiate between disulfide and thiol bonds. Field-emission scanning electron microscopy (FE-SEM) images were obtained periodically and compared with the Raman and XPS data. The Raman data suggested that the proportion of disulfide bonds before degradation was much higher in the MPDMS ONPs, and much lower in the MPMS ONPs. The FE-SEM images of the MPDMS and MPMS ONPs after 7 days of GSH incubation showed extensive MPDMS degradation, but little to no MPMS degradation, suggesting that the cleavage of disulfide bonds by GSH is responsible for the observed decomposition. The XPS data indicated that oxidized GSH (GSSG) was present on the surface of the MPDMS ONP after 7 days incubation in GSH. which This observation provides indirect evidence for reduction of disulfide bonds in MPDMS by GSH.

Doura and coworkers [82] synthesized three types of organosilica ONPs using the same thiolated MPMS and MPDMS precursors as Mekaru et al. [81]. A sol-gel NP synthesis was used to form MPDMS and MPMS ONPs, as well as an MPDMS-MPMS copolymer ONPs with varying ratios of MPDMS to MPMS. MPDMS-MPMS ONPs offer an approach to tune biodegradation, because the ratio of disulfide bonds to free thiols should be roughly proportional to the ratio of MPDMS to MPMS used to fabricate the ONPs. Raman spectroscopy, thermal gravimetric analysis, and solid-state ^13^C nuclear magnetic resonance (NMR) spectroscopy were used to determine the ratio of MPMS to MPDMS and the proportion of thiol and disulfide groups in the ONPs. Rhodamine-b dye was loaded into the ONPs and fluorescence microscopy was used to track the GSH-triggered degradation of the nanoparticle suspensions over time. Transmission electron microscopy (TEM) and SEM were used to track the degradation of the ONPs after incubation in a GSH solution for 2 to 7 days. Although only minor changes were observed in the shapes and sizes of the ONPs after this period, the SEM and TEM images showed a clearer trend after exposure to 40 mM GSH solution for 2 months: although MPMS ONPs were degraded to a negligible extent, the MPDMS ONPs were degraded completely. The extent of degradation of the MPMS-MPDMS copolymer ONPs was dependent upon the amount of MPDMS incorporated; more MPDMS resulted in more extensive degradation.

It is unclear why the MPDMS ONPs in this study did not decompose after 7 days in 10 mM GSH, given that the MPDMS ONPs synthesized by Mekaru and coworkers did decompose extensively under similar conditions. Both Mekaru and Doura used a sol-gel methodology using base hydrolysis to synthesize their MPDMS-ONPs. Studies relating various aspects of ONP synthetic methodologies to the overall disulfide content and the degradation rate would be useful in the future.

#### 3.1.4. Redox-Reversible Gelation

In a technical sense, gelation through disulfide bond formation is responsible for the mucoadhesion of the materials described in Section 3.1.2. However, mucoadhesive systems are generally governed by thiol-disulfide exchange (1 in Figure 1) and redox-based processes (2 through 10 in Figure 1) that result in the formation of new disulfide bonds between the mucoadhesive polymer and the Cys-rich mucin glycoproteins (as in Figure 9). Upon binding to a mucosal membrane, thiol- and disulfide-containing mucoadhesive materials undergo chemical reactions and gelation processes which are mechanistically different, and much more complicated than some of the redox-based examples that will be described below.

Reversible redox-triggered sol-to-gel transitions typically begin with the oxidation of a thiomer in solution to form disulfide linkages that gel the solution in situ. The thiomer is reacting with itself (either intermolecularly, or intramolecularly), as opposed to a substrate, as in the mucoadhesion examples described in Section 3.1.2. Often, the gelled networks can be reconverted to their sol phases by adding a reducing agent to break the crosslinks. This type of reaction can also be used to form more complicated networks from poly-thiolated molecules, oligomers, and/or polymers, as shown in Table 1.

**Figure 13:**
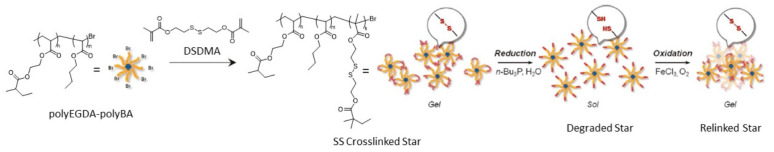
Synthetic methodology to incorporate DSDMA on polyEGDA-polyBA stars and their redox behavior. ATRP was used to synthesize polyEGDA-polyBA, generating a core-crosslinked star polymer macroinitiator (synthesis not shown). Adapted with permission from [83]. Copyright (2010) American Chemical Society.

Kamada and coworkers used core-first atom transfer radical polymerization (ATRP) to synthesize star polymers whose arms contain disulfide crosslinks (SS crosslinked star) [83]. An initial ATRP generated a star polymer macroinitiator, which consisted of a crosslinked poly(ethylene glycol diacrylate) (polyEGDA) core surrounded by linear poly(*n*-butyl acrylate) (polyBA) arms (polyEGDA-polyBA). A second chain-extension ATRP was used to incorporate bis(2-methacryloyl-oxyethyl) disulfide (DSDMA) on the ends of the star arms, forming the SS crosslinked star (Figure 13). The disulfide bonds of DSDMA were scrambled upon their incorporation into the SS crosslinked star product. Results from dynamic mechanical analysis (DMA) indicate that this process resulted in inter- and intra-molecular disulfide crosslinks between arms.

In the chain-extension ATRP step, the reaction solvent, dimethylformamide (DMF), was gelled as disulfide linkages formed. The redox responsiveness of the gels was studied in mixtures of tetrahydrofuran (THF) and chloroform (CHCl_3_). Addition of *n*-tributyl phosphine (*n*-Bu_3_P) cleaved the disulfide bonds to free thiols and converted the gel to a sol as the disulfide crosslinks were reduced at the peripheral ends of the star arms.

Kamada et al. demonstrated the reversibility of this redox cycle by reconverting the sol to a gel upon the addition of an oxidizing agent (Table 1). Comparison of the relative sizes of the peaks associated with the S-S and C-SS bonds in the Raman spectra before and after each redox step, supported the mechanistic attribution of the sol-gel transitions to redox reactions. The temperature dependence of the storage (G’) and loss (G”) moduli for the different phases was assessed by DMA. The data suggest that the original and regenerated SS crosslinked materials are structurally different. Both are different from the reduced thiol form, which has a T_g_ close to that of polyBA.

**Figure 14:**
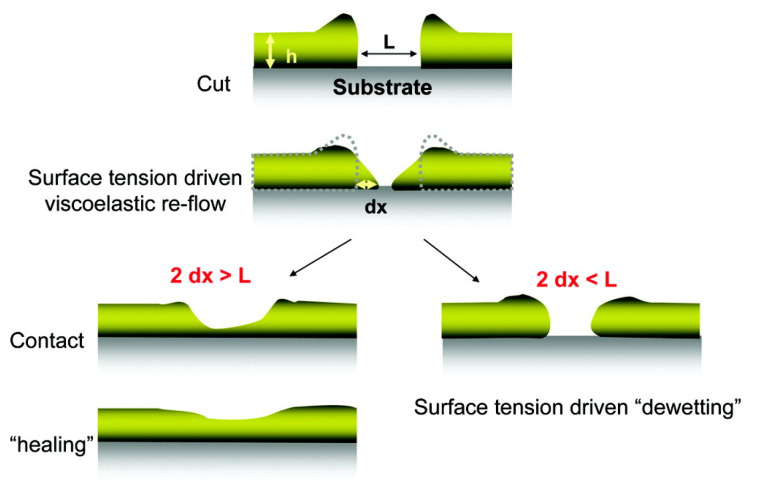
Proposed self-healing mechanism for SS crosslinked polyEGDA-polyBA stars. Reprinted with permission from [84]. Copyright (2012) American Chemical Society.

Yoon et al. studied the self-healing abilities of thin films fabricated from SS crosslinked polyEGDA-polyBA stars [84]. The mechanical properties and self-healing abilities were assessed for regenerated SS crosslinked films prepared by casting a dilute solution of reduced stars onto a silicon wafer. The kinetics of self-healing in the films was monitored after making cuts of various depths and widths using an AFM tip; the time-resolved tip-sample force vector was measured across the sample topology as the material healed at room temperature. Deep cuts that penetrated the film did not self-heal to the same extent as shallower, non-penetrating cuts. The cuts were considered “healed” when the Young’s modulus of the cut region matched that of the virgin material. Figure 14 shows the proposed self-healing mechanism.

Yoon et al. proposed that the self-healing limitations are due to the rigidity of the films: when cuts are deep and/or wide, there is insufficient flow around the fractured area to bridge the two sides of the cut. They also compared these results to the self-healing of polyEGDA-polyBA star polymers that were synthesized with a similar density of *permanent* crosslinks connecting the arms. The permanently crosslinked polyEGDA-polyBA control was not able to heal appreciably, indicating that the SS crosslinked films rely on both flow and chemical reactions between the bridged sides to self-heal. Kinetic studies, such as these, proposing molecular-level mechanisms can help assess the suitability of self-healing materials for various applications. Molecular-level self-healing processes often determine the healing efficiency because, if effective, they prevent deformations which are the source of macroscopic fracture formation [85].

**Table 1:** Redox-Reversibility in Disulfide-Crosslinked Polymers.

Polymer Backbone	Thiol or Disulfide Ligand	Gelated Solvents	Oxidizing Agent(s)	Reducing Agent(s)	Number of Reversible Cycles ^1^	Reference
Poly(acrylate)-based core-crosslinked star copolymer (polyEGDA-polyBA) (Figure 13)	DSDMA	DMF, CHCl_3_,THF	FeCl_3_ and O_2_ or I_2_ and O_2_	*n*-Bu_3_P	One full cycle	[83,84]
Branched trithiols: TMMP and TEMPIC Branched tetrathiol: PEMP Branched hexathiol: DPMP (Figure 15)	N/A, disulfides formed during oxidation of branched monomers	DMSO	DMSO or Albright-Goldman oxidation ^2^	DTT	One full cycle	[25]
Poly (2(dimethylamino) ethyl methacrylate) (Figure 17b)	1,2,3 triazole-based	Aqueous phosphate buffer	Heating in air	tris(2-carboxyethyl) phosphine hydrochloride	Five cycles	[86]
PEG functionalized chitosan (Figure 19)	BDS-functionalized or disulfide-conjugated to thiolated methyl red dye or GSH	Water	n/a, formed as disulfides	DTT	One direction	[87]
Poly(styrene-*co*-4-vinylbenzene chloride) and PEG triblock copolymer	1,2,3-triazole derivative	[EMI][TFSA] ionic liquid with DCM	Heated in air	DTT	Six redox cycles	[26,88]
Poly(benzyl ether)- PEG copolymer (ScIP) (Figure 23)	Pyridine disulfide	DMF	n/a	DTT	Non-reversible	[89]

^1^ One cycle—disulfide was reduced to the thiol; one full cycle—disulfide was reduced to the thiol and back; non-reversible—the material could not be reconverted. ^2^ Albright-Goldman oxidation conducted in DMSO with acetic anhydride.

The research of Kamada and Yoon on thiol- and disulfide-containing polyEGDA-polyBA star polymers highlights the range of applications possible for redox-reversible thiol-disulfide-containing materials. In addition, the SS crosslinked thin films were stable and self-healing [84]. However, as noted, the addition of reducing agents—such as *n*-Bu_3_P—to the SS crosslinked organogels is effective in converting them to sols [83]. Thus, thin films swollen in organic solvents or organogels could potentially be removed from surfaces in this way. This methodology would have benefits for manipulating well-defined architectures, compact structures, and site-specific functional group placements in fields as diverse as drug delivery [90,91], coatings [92], and lithography [93].

**Figure 15:**
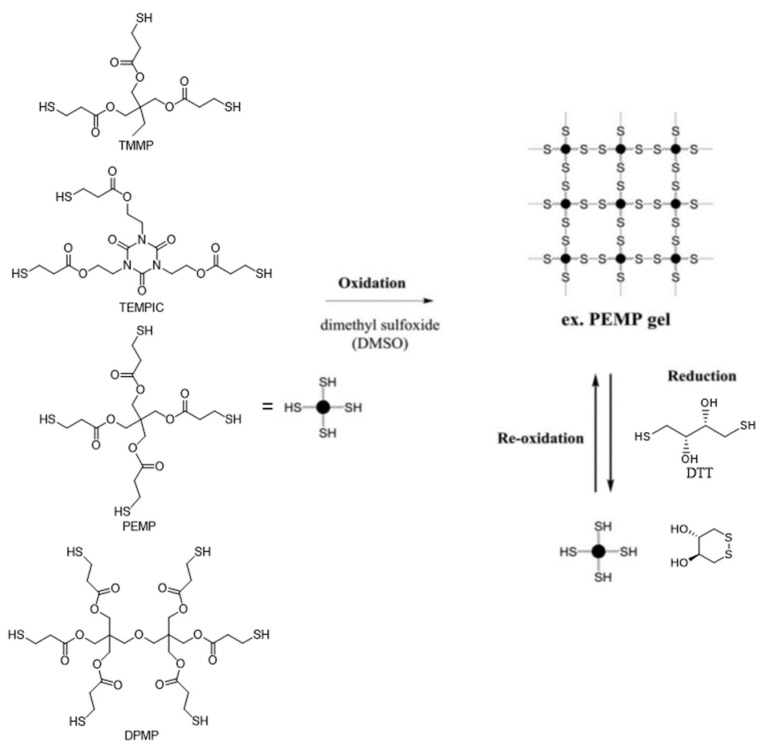
Redox behavior and synthesis of extended networks from multi-thiolated branched monomers—trimethylolpropane tris (3-mercaptopropionate) (TMMP), tris[(3-mercaptopropionyloxy-ethyl]-isocyanurate (TEMPIC), pentaerythritol tetrakis (3-mercaptopropionate) (PEMP), and dipentaerythritol hexakis (DPMP)--to generate disulfide linkages. Adapted with permission from [25]. Copyright (2017) Wiley Periodicals, Inc.

Naga and coworkers studied the redox-reversible gelation of networks formed from the crosslinking of four different multi-thiol-containing monomers with various degrees of branching: trimethylolpropane tris (3-mercaptopropionate) (TMMP), tris[(3-mercaptopropionyloxy-ethyl]-isocyanurate (TEMPIC), pentaerythritol tetrakis (3-mercaptopropionate) PEMP, and dipentaerythritol hexakis (DPMP) (Figure 15; [25]). Oxidation of the thiol-containing monomers by dimethyl sulfoxide (DMSO), which was also the solvent (Table 1), resulted in gelation.

**Figure 16:**
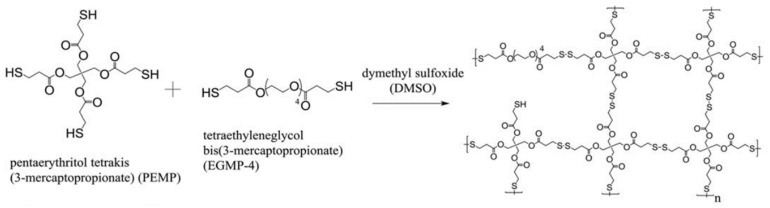
Formation of crosslinked copolymers of PEMP and EGMP-4. Adapted with permission from [25]. Copyright (2017) Wiley Periodicals, Inc.

Compression tests revealed a correlation between the degree of monomer branching (either three, four, or six), monomer concentration, and gel strength. Increasing the monomer weight percentage during synthesis, and/or decreasing the degree of monomer branching resulted in stronger organogels. The gel networks could be reconverted to sols containing the free monomers via reduction with DTT. Heating the sols at 85 °C for 8 h was sufficient to oxidize the monomers and regenerate the gels. The TMMP, PEMP, and DPMP monomers were also copolymerized with a dithiol-containing derivative of tetra ethylene glycol (EGMP-4; Figure 16). Copolymerization of the monomers with a linear dithiol produced networks with mesh sizes of about 0.5 nm.

**Figure 17:**
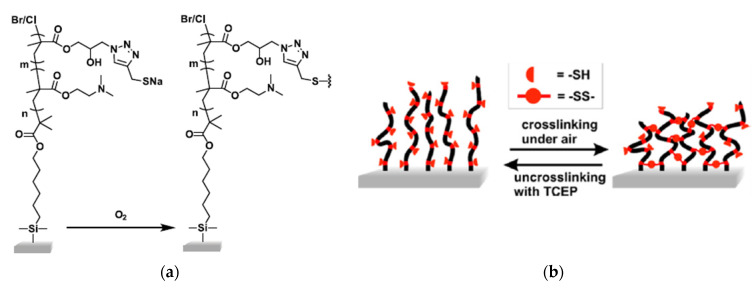
(**a**) Cartoon illustrating the reversible formation of disulfide crosslinks between polymer brush chains. (**b**) The composition of the polymer brushes studied by Mocny et al. Reprinted with permission from [86]. Copyright (2020) American Chemical Society.

Mocny and coworkers examined redox-reversible disulfide crosslinking of thiol-containing polymer brushes [86] that are thin films formed by end-grafting linear polymer chains. Unlike the sol-gel examples above, polymer brushes are grafted to a substrate, so the polymer “gels” in this case are swollen thin films attached to a substrate, and the reduction of the disulfide crosslinks merely changes the brush density, instead of forming a sol. The strength of the thin films can be correlated with brush density, which can be modified by incorporating irreversible-covalent crosslinks. Mocny et al. incorporated thiol pendant groups on poly(2-(dimethylamino)ethyl methacrylate) copolymer brushes using 1,2,3-triazole linkages in a copper(I)-catalyzed azide−alkyne cycloaddition (CuAAC) reaction (Figure 17a). The polymer brush density was reversibly changed through the oxidative crosslinking of the thiols (Figure 17b).

The swelling and dissipative properties of the polymer brushes were measured using ellipsometry and a quartz crystal microbalance, respectively, over five cycles of reduction and oxidation. Both techniques suggested that the crosslinking was reversible for two full redox cycles. Thereafter, a decrease in the crosslinking density of the oxidized form was noted. It was attributed to the irreversible formation of a small amount of non-disulfide oxidation products. This was the first study to assess the reversibility of disulfide pendant crosslinks in polymer brush films, and it demonstrates that the polymer brush densities, and consequently the swelling and dissipative properties, can be controlled in a reversible manner. Further work will be required to assess what types of polymer backbones, thiol pendant groups, and redox reagents can be combined to provide systems that are redox-reversible over many cycles. The thiol groups on the brushes studied by Mocny and coworkers were easily crosslinked by heating (60 °C). However, crosslinking a more oxidation-resistant thiol might be a better option if repeated redox cycles are desired.

Wei and coworkers also used a CuAAC reaction to tether a thiol-containing pendant group to an ABA block copolymer via a 1,2,3-triazole linkage [26,88]. The CuAAC “click” reaction is very popular for tethering thiol- or disulfide-containing groups to polymer backbones because it is efficient, functional group tolerant, and high yielding [94]. Wei and coworkers studied the redox-reversible gelation of an ionic liquid, 1-ethyl-3-methylimidazolium bis-(trifluoromethyl) sulfonyl amide ionic liquid ([EMI][TFSA]), by ABA block copolymers comprised of a thiol-containing, polystyrene A block that was insoluble in ionic liquids, and a PEG B block that was soluble in them (Table 1).

Ion gels using ionic liquids as the solvent have been proposed for use in a variety of applications, including the fabrication of electrochemical devices. These gels have been fabricated into flexible and transparent thin films with desirable properties for electronics applications because of their high ionic conductivity, low volatility, and low flammability [95,96,97]. After six gel redox cycles, the material exhibited less than 10% change in mechanical properties [26]. Sols of the ionic liquids were oxidatively crosslinked by heating, and disulfide-disulfide metathesis was proposed to be crucial to the reshaping and restructuring of the gels after reduction and re-oxidation. The same group demonstrated by mass spectrometry of small molecule analogs that disulfide-scrambling probably occurred during oxidation [88]. They suspect that residual copper salts from the CuAAC click reaction were present and catalyzed disulfide-disulfide scrambling in the molecules during oxidation. Control studies using the small molecule analogs indicated no disulfide-disulfide metathesis in the absence of the salts.

#### 3.1.5. Redox-Triggered Drug Release and Disulfide-Diselenide Chemistry

**Figure 18:**
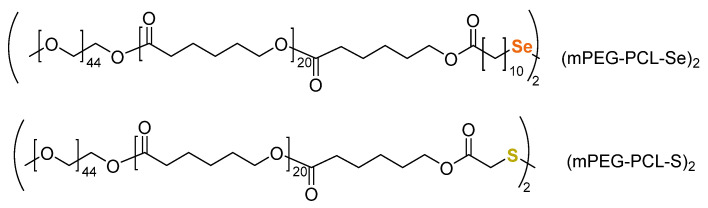
Structures of disulfide- and diselenide-linked PCL and PEG triblock copolymers synthesized by Zhang et al. [80].

Zhang and coworkers conducted redox reactions and redox reactions with triblock copolymers comprised of a hydrophobic poly(ε-caprolactone) (PCL) “B” block flanked by two hydrophilic PEG “A” blocks [80]. A disulfide or diselenide linkage present at the center of the B blocks produced copolymers of the form AB-S-S-BA or AB-Se-Se-BA, respectively (Figure 18).

The amphiphilic triblock copolymers formed micelles in phosphate-buffered saline (PBS) solutions, and micelles were formed and loaded by dialysis with the anticancer drug doxorubicin (DOX). The release of DOX from the disulfide and diselenide micelles in the buffered solution was measured with GSH after the reductive cleavage of the disulfide and diselenide bonds. The release of DOX in the buffered solution with 1% H_2_O_2_ was also measured; cancer cells are known to contain a 100-fold higher concentration of H_2_O_2_ than normal ones [98]. The non-drug loaded micelles were exposed to various concentrations GSH and the micellar polydispersity index values were calculated from dynamic light scattering measurements.

As the disulfide and diselenide bonds break in the hydrophobic core, the micelles dissociate or change size. Thus, the polydispersity increases can be correlated with the degree of S-S or Se-Se bond cleavage. When exposed to 1 mM GSH, the polydispersity of diselenide micelles changes more rapidly, and increases to a greater extent, when compared with the disulfide micelles. This result indicates that Se-Se bond scission in the diselenide micelles occurs more rapidly and to a greater extent than S-S bond scission in the disulfide micelles. Solutions of disulfide and diselenide micelles were incubated with GSH at concentrations ranging from 20 μM to 2 mM. The two micelles exhibited comparable minor changes in polydispersity after being incubated for 6 h in 20 µM GSH. Conversely, after 6 h of exposure to 1 or 2 mM GSH, the diselenide micelle polydispersity increased much more than that of the disulfide micelles.

Fluorescence spectroscopy was used to quantify the DOX release from the micelles as a function of time in a simulated blood environment with and without 10 mM GSH or 1% H_2_O_2_. The results demonstrated that the diselenide-linked micelles were more responsive to both redox conditions, releasing DOX more rapidly, and to a greater extent, than the disulfide-linked micelles. Disulfide micelles exposed to 1% H_2_O_2_ in PBS at 37 °C for 32 h released the same percentage of DOX (40%) as the control sample (in PBS at 37 °C). However, the diselenide micelles released about 65% of the loaded DOX under the same oxidizing conditions, which was slightly greater than the 60% lost by the control sample in PBS (that is, without H_2_O_2_). After 32 h of exposure to 10 mM GSH in PBS at 37 °C, the DOX-loaded diselenide micelles had released about 90% of the initial amount of DOX, whereas the disulfide micelles released about 70%. However, the difference between the mass of DOX released from disulfide and diselenide micelles upon exposure to GSH or H_2_O_2_ is even greater than the difference based on the percentages because more DOX can be loaded into the diselenide micelles. The DOX drug loading content was greater for the diselenide micelles, possibly due to coordination between Se and the quinonyl ring of DOX [80]. In this regard, diselenide-linked micelles may be more effective for triggered anti-cancer drug release for types of cancer associated with relatively lower GSH concentrations, such as Hela, B16F10, and 4T1 cells [98]. The concentration gradient between these types of cancer cells and surrounding non-cancerous cells is lower, relative to many other cancer cell types, which commonly have GSH concentrations closer to 10 mM [99]. The diselenide-linked micelles are more responsive to GSH concentration changes in the low mM range, which suggests that they may be more effective for anti-cancer drug delivery for types of cancers associated with GSH concentrations in the low mM range. Lang et al. [100] also found that DOX-loaded diselenide-linked poly(ester)-*co*-poly(urethane) micelles were more sensitive to reduction, and had better antitumor activity than the analogous disulfide-linked micelles. Although disulfides are well suited for use in the body, due to the ubiquity of thiol and disulfide groups in nature, diselenides can undergo some of the same reactions as disulfides, and studies comparing the two may reveal possible niche applications for diselenide chemistry. Self-healing diselenide systems have been reported as well [6]. Recently, Perera et al. demonstrated that norbornene-terminated poly(ethylene glycol) and poly(2-hydroxypropyl methacrylate-stat-mercaptoethyl acrylate) polymeric hydrogels could participate in seleno-sulfide exchange reactions with 5,5′-diselenide-bis(2-aminobenzoic acid) upon irradiation with UV light, resulting in reversible hydrogel softening and stiffening [101]. Unlike the redox-reversible systems in Section 3.1.4, which can undergo reversible sol-to-gel transitions in response to a redox stimulus, the hydrogel system described by Perera and coworkers can alter its stiffness under irradiation via seleno-sulfide exchange.

Several additional examples of disulfide-based drug delivery systems will be considered in Section 3.1.6 and Section 3.1.7. However, the handful of examples covered here is a minute subset of the literature since disulfide-linked drugs were first approved for human use in the early 2000s [12]. As shown in Figure 3, there is a wide variety of disulfide-based drug delivery systems for nucleic acid-based drug delivery [10]. For more information, please consult reviews by Dutta, Saitoa, Quinn, and Wang [10,11,12]; as well as several reviews on redox-responsive [9,102,103,104,105] or multi stimulus-responsive [15] drug delivery systems, in addition to general reviews on them [8,79,106].

#### 3.1.6. Loading Small Molecule Cargo into Networks

Thiol-disulfide exchange (1 in Figure 1) and redox reactions (2 through 10 in Figure 1) can be used to generate mixed disulfides, R’SSR, where R’ is a polymer backbone and R is a small “cargo” molecule derived from a thiol-containing precursor. Thiol- and disulfide-containing polymers loaded with ‘cargo’ have been explored as drug delivery systems, sensors, and as analytical tools. The chemical reactions that govern a small molecule’s reversible conjugation to—and subsequent release from—an extended network are the same as those mentioned in preceding sections. In comparison to redox-reversible gel systems, cargo binding systems can bind cargo to a site in the network via a disulfide linkage. In the following examples, disulfide bonds are used to regulate the binding of small molecules to a network, as opposed to regulating the crosslinking between and within an extended network.

**Figure 19:**
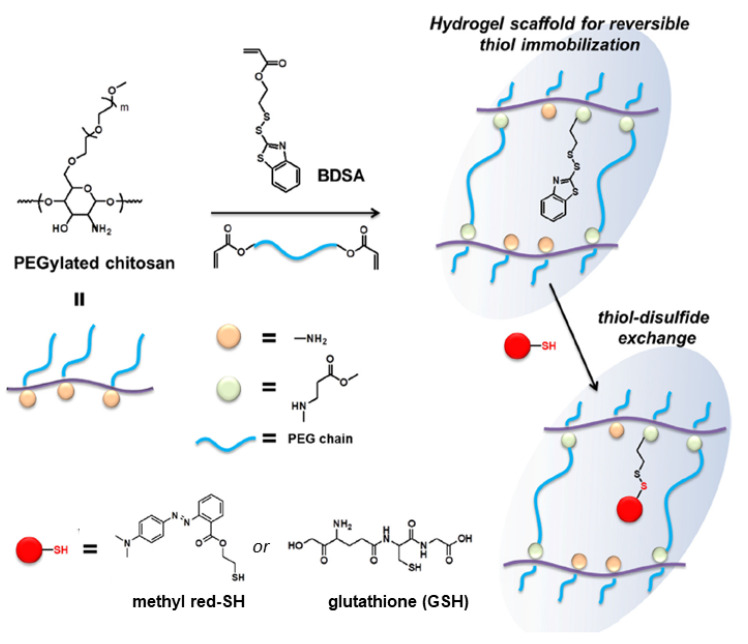
Conjugation of BDSA to PEG functionalized chitosan via an aza-Michael addition. A hydrogel was formed from the BDS-containing PEG functionalized chitosan, and a thiol-disulfide exchange reaction tethered the thiolated cargo molecules (red dot) to it. Adapted with permission from [87]. Copyright (2020) Elsevier B.V.

Arslan and coworkers synthesized a disulfide-containing poly(ethylene glycol) (PEG) functionalized chitosan (Figure 19; [105]). Amine groups were reacted in an aza-Michael addition with benzothiazole disulfide acrylate (BDSA), in the presence of poly(ethylene glycol) diacrylate (PEGDA), to furnish benzothiazole disulfide (BDS)-containing pendant groups and form permanent crosslinks between the PEGDA and the chitosan chains. Most thiolated small molecules readily exchange with BDS groups. In the hydrogel states of the PEG functionalized chitosan, thiol-disulfide exchange reactions were conducted between the BDSA and either thiolated methyl red dye or GSH. The thiol-disulfide exchange reactions were used to incorporate the methyl red dye or GSH into the hydrogel network via disulfide bonds.

As will be discussed in Section 3.1.7, disulfide bonds can crosslink polymer networks and bind small cargo molecules to the networks. The resulting materials are capable of simultaneously being degraded and releasing cargo in response to a trigger, such as a reducing agent.

The cargo molecules released from networks can also be used as sensors. For example, the amount of released cargo can be related to the amount of analyte that has reacted in an exchange reaction. Although this subject is outside the scope of this review, it is sufficiently related to warrant some mention. Thus, Tomei and coworkers recently fabricated filter paper-based electrochemical cells that can detect as little as 60 μM concentrations of GSH in blood. The strips detect cysteamine (a thiol) as a product of the thiol-disulfide exchange reaction with GSH based on selective oxidation at a carbon black/Prussian blue electrode in the strips [18].

**Figure 20:**
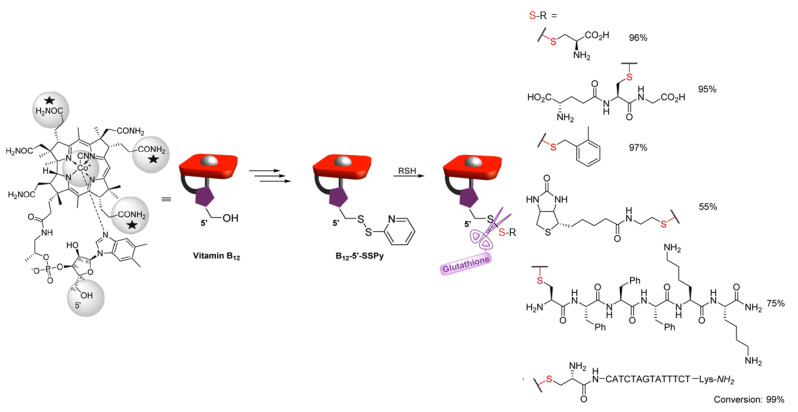
Synthesis of the pyridyl disulfide derivative of B_12_ (B_12_-5′-SSPy). Thiols were reacted with B_12_-5′-SSPy to form a variety of disulfide-linked conjugates, B12-SS-R. The structure at bottom-left is a synthetic nucleic acid analog. Adapted with permission from [107]. Copyright (2016) American Chemical Society.

Wierzba and coworkers designed and synthesized a vitamin B_12_ (B_12_) derivative for direct conjugation to small molecules or polymer networks via a disulfide linkage (Figure 20; [107]). B_12_ has been touted as a potential transport agent for drug delivery and in vivo imaging agents because of its high affinity toward transport proteins, and its propensity to be accumulated in dividing cells [108,109,110,111]. B_12_ has a unique dietary uptake pathway, and the incorporation of B_12_ groups into a drug delivery system can improve the efficacy of drug delivery by expanding the metabolic pathways to include B_12_ uptake.

Although other metabolically stable B_12_ derivatives have been synthesized, they do not always retain a high affinity for transport proteins. The domains of B_12_ circled in gray in Figure 20 have been explored as conjugation sites. Amide-based conjugates formed at the starred positions still demonstrate a strong binding affinity for trafficking proteins [108]. Most conjugates are formed with ester, amide, or carbamate linkages [107], which—unlike disulfide bonds—are not cleavable in cells.

The same authors also developed a B_12_ derivative based on disulfide conjugation at the 5′ position of the ring. A pyridyl disulfide precursor (B_12_-5′-SSPy) was found to be highly reactive toward a variety of thiols in thiol-disulfide exchange reactions (Figure 20). Like benzothiazole disulfides, pyridyl disulfides are highly reactive in thiol-disulfide exchange reactions. Thus, they are excellent precursors of unsymmetrical disulfides used in cargo tethering applications, including cyclic peptide conjugates [112]. The addition of glutathione (GSH) was shown to result in disulfide bond scission for the R groups examined. In theory, when both B_12_ and a drug are conjugated to a network via disulfide linkages, GSH-induced reduction can be used to trigger concomitant release of the drug and B_12_.

The research of Wierzba et al. provides an alternative perspective for the release of cargo molecules. Research focusing on synthetic modifications of molecules prior to conjugation would expand the range of cargo capable of being used in disulfide-based drug delivery systems. Despite the effects of enhanced permeability and retention, which are afforded to any nanosized anti-cancer drug delivery system, disulfide-based nano-drug delivery systems still suffer from inefficient delivery and cellular uptake [11]. However, the quality of drug delivery and release can be enhanced by the addition of various biological targeting groups, such as folic acid, biotin, and galactose.

#### 3.1.7. Thiol-Disulfide Exchange and Redox Reactions That Initiate Degradation or Cascade Reactions

Several applications have been proposed for thiomers or disulfide-containing polymers that can be triggered to react in a cascade-like fashion, after an initial thiol-disulfide exchange (1 in Figure 1) or redox reaction (2 through 10 in Figure 1) occurs. Examples include reactions that ultimately result in the formation of new bonds, or the destruction of bonds in self-immolative (i.e., degradation) processes. Disulfide- and thiol-containing polymers offer mild, yet targeted opportunities to initiate and trigger the cascade.

Self-immolative polymers have been proposed for addressing several biomedical and environmental problems. They include the formulation or fabrication of biomedical materials, drug delivery systems, tissue scaffoldings, biodegradable polymers, soil treatments and microelectronics [113,114,115,116]. Functional groups containing cleavable bonds, such as esters, amides, polysulfides, etc., have been used for self-immolative polymer formulations [117]. In that regard, the inherent biological redox responsiveness of disulfide-containing polymeric networks makes them ideal for self-immolative polymers for environmental and biomedical degradation applications.

Disulfide-linked networks may degrade by two basic scenarios. In the simpler case, disulfide linkages functioning as crosslink or branch sites, can be cleaved to cause network decomposition into constituent monomers, oligomers, and/or sub-branches upon exposure to a stimulus. Such a case was described by Naga and coworkers [25] (Section 3.1.4). None of their examples, however, achieved redox-triggered decomposition of linear polymers into constituent monomers.

More mechanistically complex degradation pathways for self-immolative polymers occur in the second scenario. In these systems, disulfide bond scission leads to a cascade reaction that results in the subsequent cleavage of other, non-disulfide, linkages in the polymer. Polymers linked to pendant groups via disulfide bonds, as well as systems with disulfide linkages along the main polymer backbone, can exhibit true self-immolation. Generally, these systems have well-defined architectures. Commonly, the cleavage of a disulfide bond results in a cascade decomposition process that affects adjacent groups which have labile bonds, such as ester linkages. If disulfide bonds are used to crosslink a network and tether cargo to it, simultaneous drug delivery and decomposition (into monomers) can be achieved under appropriate conditions. The biodegradability, biocompatibility, and environmental impact of the decomposition products are key factors to consider in this regard, as are the decomposition mechanisms which are consequential in in vivo applications.

#### Cascade Reactions Triggering Decomposition

**Figure 21:**
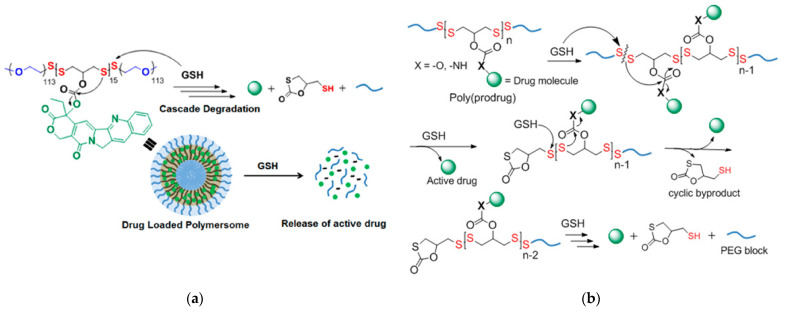
(**a**) GSH-induced cascade degradation of polydisulfide-CPT polymersomes. (**b**) Proposed mechanism for the degradation of polydisulfide-CPT by GSH. Adapted with permission from [118]. Copyright (2019) American Chemical Society.

Bej and coworkers [118] used a polycondensation reaction between a hydroxyl-containing dithiol and pyridyl disulfide to create a poly(disulfide) with a free hydroxyl group on each monomer unit. Post-polymerization modification was used to incorporate the anticancer drug camptothecin (CPT) via an ester linkage. Then, a thiol-disulfide exchange between thiol-terminated PEG oligomers and terminal pyridyl disulfides of the polydisulfide was used to generate an amphiphilic ABA triblock copolymer prodrug (polydisulfide-CPT). The polydisulfide-CPT contained two disulfide bonds per polydisulfide monomer directly along the polymer backbone (Figure 21a). The polydisulfide-CPT formed polymersomes in aqueous solutions, and exposure to 10 mM GSH resulted in the release of CPT and polymer backbone degradation. In the proposed mechanism (Figure 21b), initial GSH-triggered disulfide bond scission breaks the triblock copolymer into constituent PEG and polydisulfide blocks and generates a free thiol group in the PDS block. The free thiol then participates in an intrachain nucleophilic attack of the polydisulfide-CPT ester linkage, resulting in bond scission and CPT drug release.

Comparative studies among the polydisulfide-CPT, free CPT, and an analogous CPT-free triblock copolymer were conducted to gauge the degree of cellular internalization, toxicity to HeLa cancer cells, and toxicity to non-cancerous cells. Based on the half-maximal inhibitory concentration (IC_50_) values (3.1 and 5.7 μg/mL, respectively), treatment of the cancer cells with polydisulfide-CPT was more effective than treatment with free CPT. Additionally, after 36 h exposure to 300 μg/mL (based on CPT content), polydisulfide-CPT killed < 25% and free CPT killed > 60% of the non-cancer cells. Because the non-CPT containing polymer exhibited negligible toxicity toward both cancer and non-cancer cell lines, the undesired toxicity of polydisulfide-CPT in normal cells appears to be due to the presence of CPT, rather than the polymer backbone. By extension, the greater toxicity of polydisulfide-CPT towards the cancer cells can be attributed to the in situ release of CPT, and not the polymer backbone blocks themselves.

Yin and coworkers [19] designed and synthesized PEG and poly(methyl methacrylate) (PMA) diblock copolymers (BCPs) conjugated to CPT as dual-responsive micellar drug delivery systems (GR-BCPs). The GR-BCPs were designed for the dual-responsive release of CPT in response to elevated concentrations of reactive oxygen species (ROS) and/or GSH (i.e., concentrations found in cancer cells). The CPT was conjugated to the PMA block of the BCPs via a labile thioether bond that was cleavable in the presence of excess ROS or glutathione. The GR-BCPs incorporated disulfide linkages that were designed to be cleaved in the presence of high concentrations of GSH, as well as thioketal bonds that were designed to be cleaved in the presence of high concentrations of ROS. Single-responsive BCPs were also prepared with either disulfide bonds (G-BCPs) selective for elevated GSH concentrations or thioketal bonds (R-BCPs) for response to elevated ROS concentrations.

Dual-responsive systems designed to respond to the presence of either GSH or ROS can enhance drug release because overexpression of ROS and GSH occur non-homogenously within cancer cells [119]. Although it is possible to design successful drug delivery systems for the precise targeting of specific cellular regions—such as mitochondria, which are known to overexpress ROSs—this approach requires the design and synthesis of extremely complex drug delivery systems [120]. The observation by Yin et al. that GR-BCP micelles can enter all parts of the cell suggests that CMT can be released in any area where either ROS or GSH is sufficiently overexpressed.

**Figure 22:**
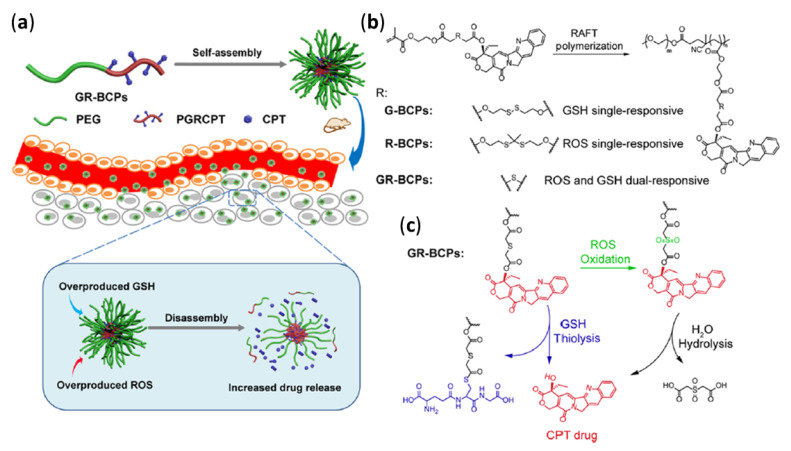
(**a**) Self-assembly of GSH- and ROH-dual-responsive BCPs (GR-BCPs) comprised of a PEG block and CPT-PMA conjugate block (PGRCPT; top). Disassembly of GR-BCP with subsequent CPT drug release in mouse tumor tissue (bottom). (**b**) Reversible addition-fragmentation chain transfer (RAFT) copolymerization of PGRCPT and PEG blocks, and structures of R groups for dual-responsive BCPs (GR-BCPs), and single-responsive BCPs that respond to GSH (G-BCP) or ROS (R-BCP). (**c**) GR-BCP structure before and after subsequent disassembly and CPT release for GSH- and RSH-triggered pathways. Reprinted with permission from [19]. Copyright (2020) American Chemical Society.

All three of the BCPs shown in Figure 22 formed nano-sized micelles in PBS solution. The release of CPT from the three micelles was studied in vitro, using HeLa cancer cells, as well as in vivo, by treating mice with tumors with intravenous BCP solution injections. The dual-responsive GR-BCP exhibited the best performance in vivo and in vitro: the IC_50_ values were 6.3 μM for BR-BCP, 17.8 μM for G-BCP, and 28.9 μM for R-BCP. The increase in mouse tumor size was smallest in mice injected with GR-BCP.

Although drug release in response to stimuli associated with intracellular redox chemicals has been widely studied, other stimuli can function as secondary triggers in multi stimulus-responsive drug delivery systems. In fact, any other differences between the microenvironments of cancerous and non-cancerous cells (e.g., pH, concentrations of specific enzymes, and oxygen levels (due to hypoxia) [121]), can, in principle, be exploited for selective drug release.

**Figure 23:**
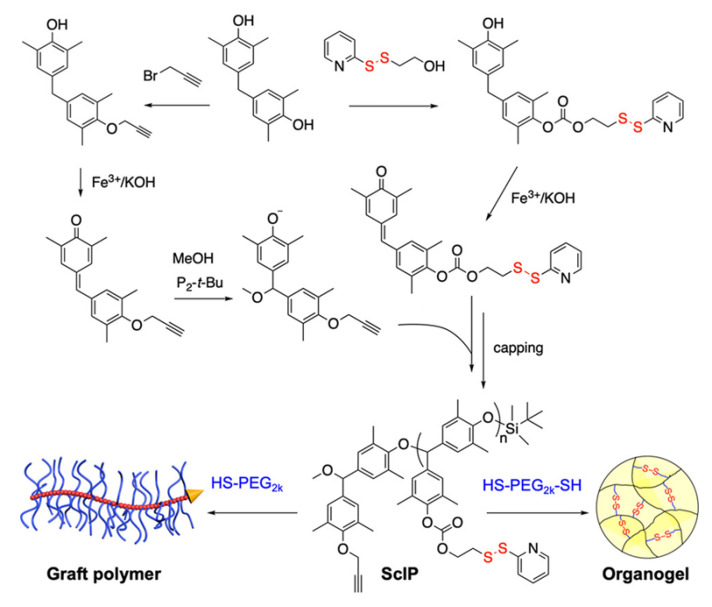
Synthesis of PBE-based ScIPs bearing pyridyl disulfide pendant groups, and their reaction with mono-or bis-mercapto-terminated PEG to form PEG-grafted ScIPs (ScIP-*g*-PEG) or organogel networks with PEG-disulfide crosslinks. Adapted with permission from [89]. Copyright (2019) American Chemical Society.

**Figure 24:**
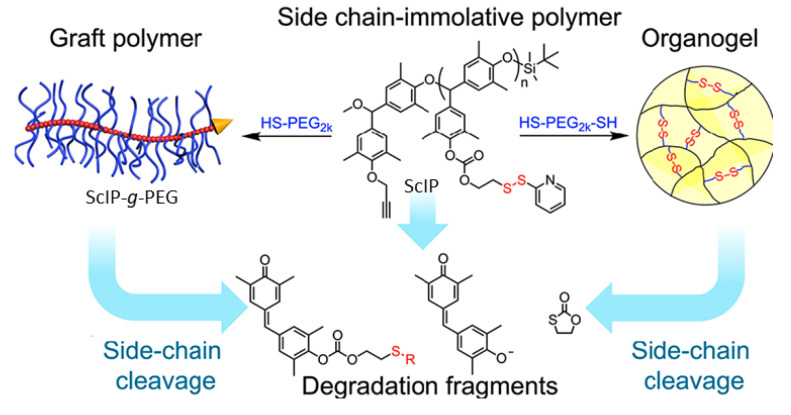
PBE-based ScIP structure, and reactions with mono- and bis-mercapto-terminated PEG. The graft polymer (ScIP-g-PEG), organogel, and ScIP can all be degraded by DTT. Reprinted with permission from [89]. Copyright (2019) American Chemical Society.

Xiao and coworkers [89] placed pyridyl disulfide-containing pendant groups on poly(benzyl ether) (PBE) backbones to form side chain-immolative polymers (ScIPs; Figure 23) which can be degraded upon exposure to reducing agents, such as DTT. These pendant groups were then reacted in a thiol-disulfide exchange reaction with either a mono-mercapto- terminated PEG (HS-PEG) or a bis-mercapto-terminated PEG (HS-PEG-HS), to produce a PEG-grafted ScIP (ScIP-g-PEG) or an extended network crosslinked by PEG groups via disulfide bonds (Figure 24).

**Figure 25:**
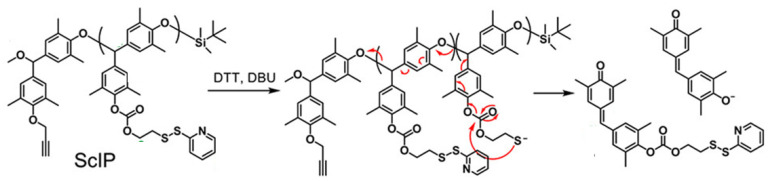
Proposed mechanism for the unidirectional self-immolative depolymerization of the ScIP after exposure to DTT and DBU. Data from electrospray ionization-mass spectrometry were able to identify the two molecules on the far right as the predominant products Reprinted with permission from [89]. Copyright (2019) American Chemical Society.

The self-immolative behavior was studied in the solution, organogel, and solid states by gel permeation chromatography, ^1^H NMR, electrospray ionization-mass spectrometry, and molecular modeling. The data indicated that exposure of the ScIPs (in the solid and solution states) to DTT and 1,8-diazobicyclo [5.4.0]undec-7-ene (DBU) resulted in unidirectional self-immolation, triggered by DTT-induced cleavage of the pyridyl disulfide bonds (Figure 25)**.**

Self-immolation in this system is proposed to begin with DTT-triggered pyridyl disulfide bond rupture, which generates a sulfhydryl anion capable of backbiting the carbonate group and generating a phenolate ion. Phenolate ions are known to trigger self-immolation of PBEs when initiated from an end group. Since the PBE-based ScIPs decompose from their pendant groups, full decomposition via depolymerization occurs unidirectionally along one side of the ScIP, producing monomers. The other side of the ScIP remains intact, as an oligomer. Because the pyridyl disulfide pendants on the intact oligomer continue to react with DTT over time, so the ScIP can be depolymerized almost completely into constituent monomers or small oligomers.

**Figure 26:**
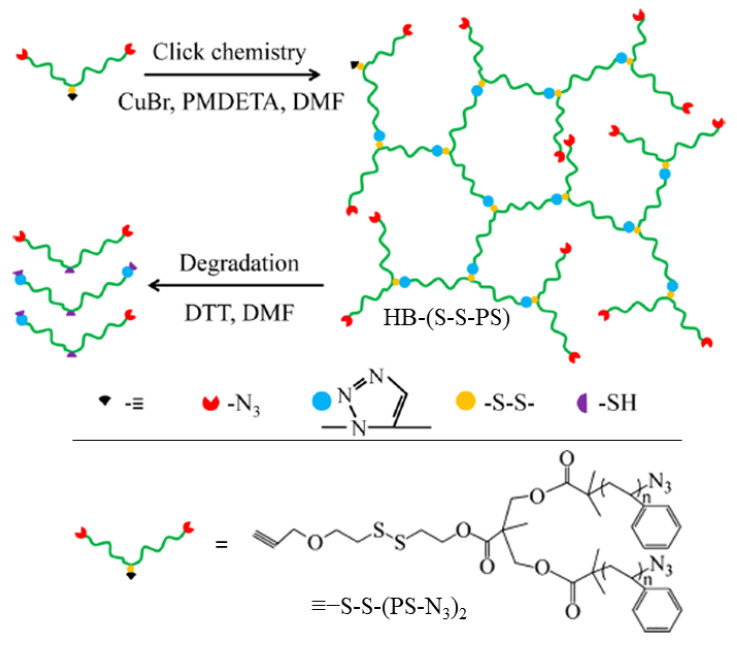
Structure and reaction of the seesaw-like macroinitiator (≡−S-S-(PS-N_3_)_2_) used to form HB-(S-S-PS), and by-products of the DTT-induced degradation of HB-(S-S-PS). Adapted with permission from [122]. Copyright (2014) American Chemical Society.

Li et al. [122] synthesized disulfide-linked hyperbranched polystyrene (PS) networks (HB-(S-S-PS)) with well-defined architectures. “Seesaw”-like macroinitiators were prepared wherein dual azide-terminated PS pendant groups branched from a middle region that contained a disulfide linkage and a terminal alkyne (≡−S-S-(PS-N_3_)_2_) (Figure 26). Hyperbranched networks were formed by reacting the PS-chains in a CuAAC click reaction (Figure 26). The objective was to synthesize a well-defined hyperbranched polymer with disulfide linkages as a “model”. The disulfide-linked hyperbranched polymers reported in previous literature lacked the well-defined architectures that are better for biomedical applications [123,124,125,126,127]

The seesaw macroinitiator was designed so that a CuAAC could be used to form the network, ensuring high specificity during branch formation. The DTT-induced cleavage of the disulfide bonds was monitored by DLS. Data from kinetic studies conducted in DMF solutions suggest that the degradation of HB-(S-S-PS) by DTT proceeds by both faster (in peripheral regions) and slower (in interior regions) reaction pathways. The interior disulfides react more slowly due to greater steric hinderance, restricted chain mobility, and lowered reagent accessibility. Both reactions exhibited first-order dependence of on DTT concentration [128,129,130], but both rate constants are several orders of magnitude lower than those reported for the reactions of DTT with small molecule thiols [131].

**Figure 27:**
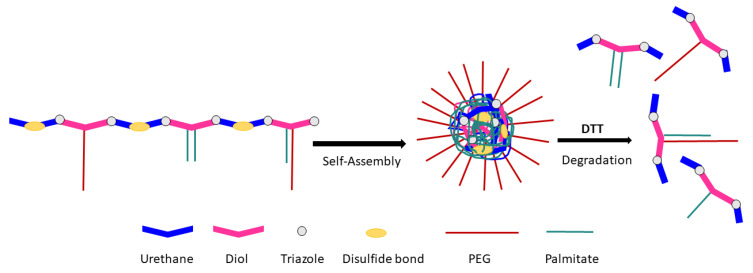
PEG-grafted PU amphiphilic polymers made by Zhang et al. [132]. The main chains are cleavable upon reduction.

Zhang and coworkers [132] took a similar approach when synthesizing amphiphilic PEG- and palmitate-grafted linear poly(urethane)s (PUs), with disulfide linkages along the main chain (Figure 27). The amphiphilic polymers formed micelles in solution that were degradable upon being reduced.

#### Cascade Reactions Resulting in Polymerization or Material Rearrangements

**Figure 28:**
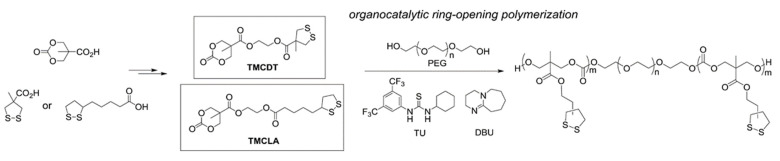
Synthesis of TMCDT and TMCLA, and the ring opening polymerization to form PEG-PC ABA triblock copolymers with 1,2-dithiolane pendant groups. The block copolymer product on the right is for the ring opening polymerization of TMCDT with PEG. Reprinted with permission from [31]. Copyright (2017) American Chemical Society.

Zhang and Waymouth [31] synthesized ABA triblock copolymers comprised of PEG and polycarbonate (PC), the latter of which was appended with 1,2-dithiolane pendant groups. Two different 1,2-dithiolane containing molecules, TMCDT and TMCLA, derived from methyl asparagusic acid and lipoic acid, respectively, were synthesized (Figure 28). Triblock copolymers incorporating either TMCDT, TMCLA, or both were synthesized by the ring opening polymerization (ROP) of the cyclic carbonate groups.

**Figure 29:**
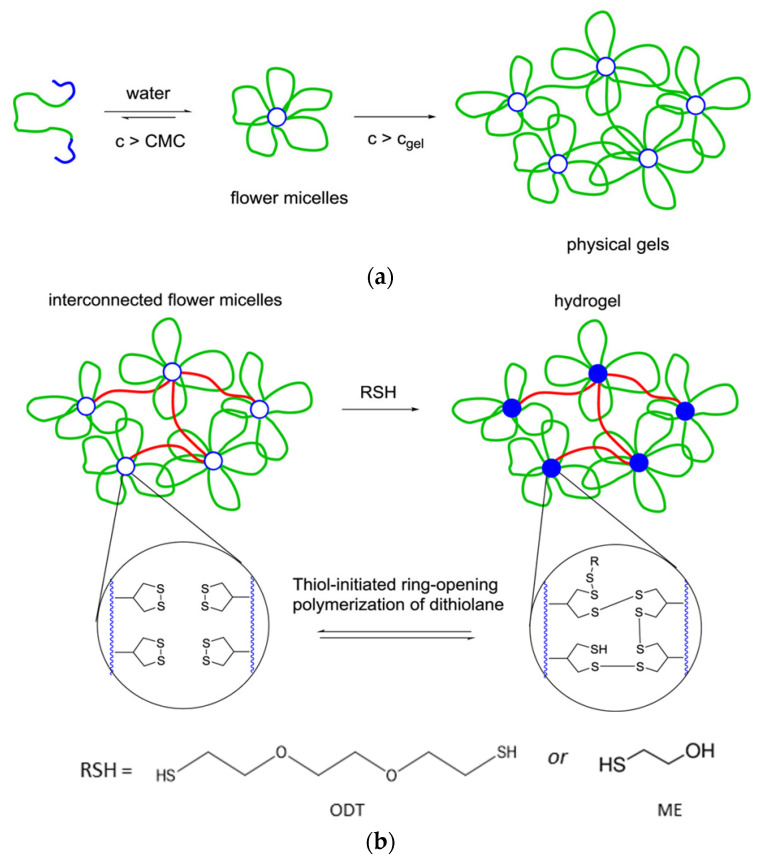
(**a**) ABA triblock copolymers, like DBCP, with hydrophobic end blocks and their self-assembly into flower micelles above their critical micelle concentrations and gelation at still higher concentrations. (**b**) Formation of chemically crosslinked gels from DBCPs after addition of dithiol 3,6-dioxa-1,8-octadecanethiol. Adapted with permission from [31]. Copyright (2017) American Chemical Society.

In aqueous solution, both 1,2-ditholane-functionalized block copolymers (DBCPs) formed micelles above their critical micelle concentrations, and further aggregated at higher concentrations. Eventually, they formed physical gels at ~10–15 wt% concentrations. This type of concentration dependence is well-known for ABA triblock copolymers with hydrophobic end blocks (Figure 29a) [133,134,135,136,137].

**Figure 30:**
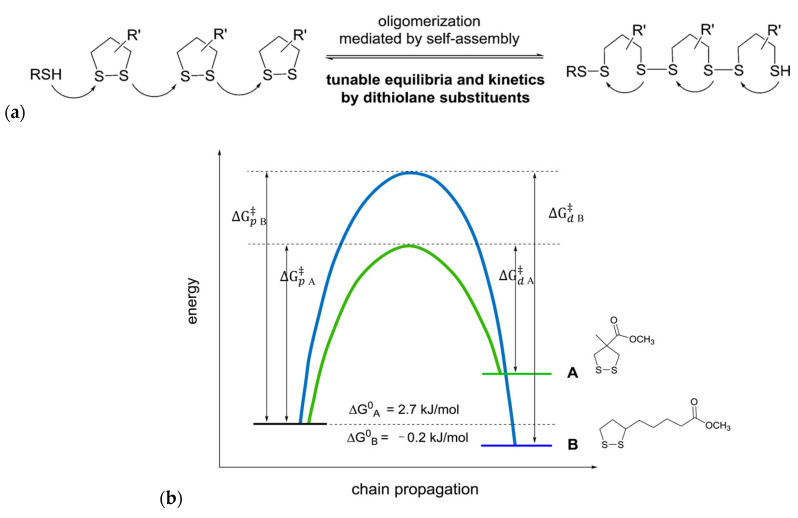
(**a**) Ring opening polymerization of 1,2-ditholane pendant group in the hydrophobic micelle cores upon exposure to a small molecule thiol. (**b**) Ring opening polymerization energy profiles (relative) for TMCLA (green) and TMCDT (blue) monomers. Reprinted with permission from [31]. Copyright (2014) American Chemical Society.

To crosslink chemically the DBCP micelles and form a hydrogel, a dithiol, 3,6-dioxa-1,8-octadecanethiol (ODT), was added to solutions of the DBCP at concentrations above the critical micelle concentration. Zhang and Waymouth found that hydrogelation occurred when even a monothiol, 2-mercaptoethanol (ME), was added. Furthermore, the observation that DBCP hydrogels formed with ODT were physically and rheologically similar to those formed with ME indicates that gelation was a result of a thiol-initiated ring opening polymerization (Figure 29b and Figure 30a).

Unlike the physically crosslinked gels, the chemically crosslinked TMCDT- and TMCLA-based DBCP hydrogels were rheologically dissimilar. The chemically crosslinked TMCDT-based DBCP hydrogels were structurally dynamic, flowed under applied stress, and exhibited self-healing properties while the TMCLA-based hydrogels were rigid, not moldable, not deformable, and not self-healing. Gels from the mixed TMCDT-TMCLA DBCPs exhibited intermediate rheological properties: higher TMCLA contents led to more brittle and more rigid gels. Gelation was reversible and tunable based on temperature, polymer concentration, and pH.

Thermodynamic and kinetic parameters associated with the ring opening polymerization of TMCLA and TMCDT were determined and used to construct the energy profile in Figure 30b. It suggests that the ring opening polymerization of TMCDT is less favored thermodynamically than that of TMCLA, but it proceeds with a lower energy barrier. The authors proposed that TMCLA hydrogels are more dynamic than the rigid TMCDT gels because TMCLA can crosslink (via ring opening polymerization) and un-crosslink (via depolymerization) much more rapidly than TMCDT.

Studies in non-aqueous solutions demonstrated the role of self-aggregation to the ring opening polymerization-induced crosslinking. Thus, the DBCPs were unable to crosslink in acetone; an initial self-assembly step is required to crosslink the DBCP micelle cores.

**Figure 31:**
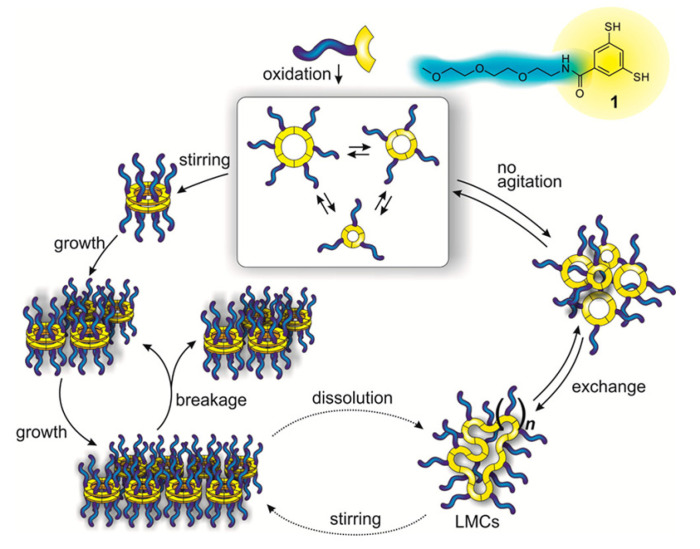
Oxidation and subsequent self-assembly of small dithiol amphiphiles with and without stirring. Reprinted with permission from [138]. Copyright (2017) American Chemical Society.

Komaromy et al. [138] investigated the role of self-assembly on the distribution of oxidation products formed by small, amphiphilic dithiol building blocks. They showed that controlling the self-assembly pathway can control covalent disulfide bond formation. From analyses by ultra high-performance chromatography-mass spectrometry (UPHC-MS), it was possible to detect the influence of several experimental variables (including stirring) on the ring sizes which ranged from trimers to 51-mers (Figure 31).

**Figure 32:**
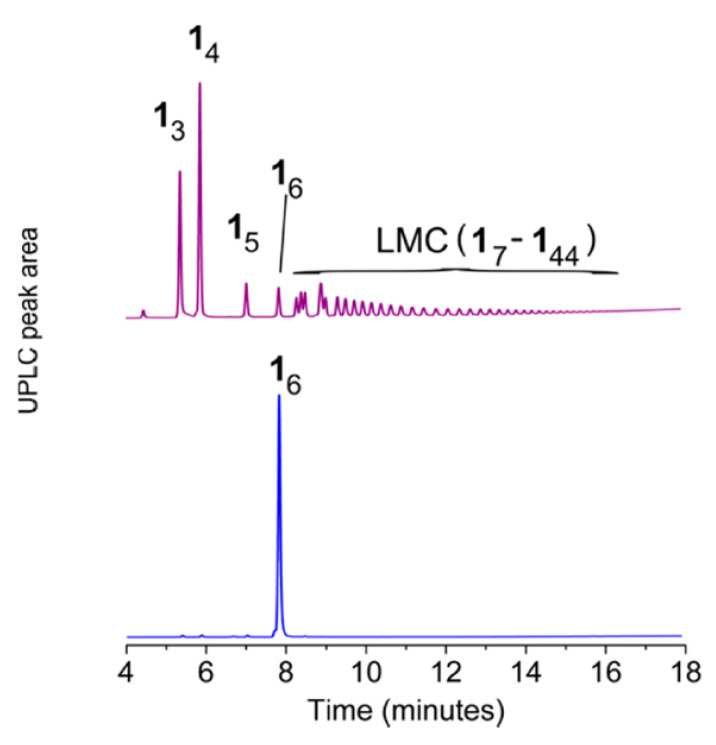
Mass spectra of dithiol amphiphiles from unstirred (**top**) and stirred (**bottom**) solutions after exposure to an oxidant for one week. Reprinted with permission from [138]. Copyright (2017) American Chemical Society.

The role of the self-assembly process on oxidation product specificity was examined using stirred and un-stirred reaction conditions with different co-solvents. Some conditions generated one ring type with high specificity, whereas others generated a diverse array of different ring sizes. For example, after stirring for 7 days, the mass spectrum of the reaction mixture in a 9:1 aqueous borate buffer:DMF solution showed thiol oxidation products that included a trimer, a tetramer, and a distribution of large macrocyclic rings (LMCs; Figure 32, top). When unstirred, the reaction mixture produced hexamers exclusively (Figure 32, bottom)! These results demonstrate that self-assembly can dictate the type and nature of disulfides formed during the oxidation of multi-thiolated amphiphiles in solution. The highly specific production of the hexamers in the stirred solution is an example of exclusive and autocatalytic formation of one dynamic combinatorial library member. Additionally, because any examples of disulfide-containing micellar drug delivery systems have been reported, the role of self-assembly on their reactivity may be important when considering long-term storage and drug delivery.

### 3.2. Disulfide-Disulfide Metathesis-Based Systems

#### 3.2.1. Introduction to Self-Healing Materials and Current Challenges in the Field

Many examples of disulfide-based self-healing polymeric systems, including gels and elastomers, have been reported [4,5,6]. Here, we review briefly research that is historically important and is currently being conducted to address the biggest challenges facing the development of new disulfide-based self-healing polymers.

Self-healing polymers were reported first in the 1970s [139,140,141]. In the early examples, the self-healing was driven by chain interdiffusion, which occurs at temperatures above the T_g_ [6,142]. A recent review by Utrera-Barrios et al. [6] identified four generations of self-healing polymers, beginning with the development of extrinsic self-healing materials in the early 2000s. ‘Extrinsic’ self-healing materials include encapsulated healing reagents that are released upon fracture to heal the material. Additionally developed in the early 2000s, second generation ‘intrinsic’ self-healing materials do not require additional reagents, although external stimuli are sometimes added to speed self-healing.

Some intrinsically self-healing systems incorporate dynamic covalent bonds, such as disulfide, diselenide, ditelluride, alkoxyamine, oxime-carbamate, urea, imine, and boron-based bonds [5,6]. Intrinsic self-healing prompted by reversible reactions, such as transesterification or Diels-Alder reactions, has also been explored. Intrinsic self-healing can also be achieved by strong non-covalent interactions, including dipole-dipole, hydrogen bonding, host-guest, metal-ligand, ionic interactions, and even shape memory responsiveness.

It is challenging to find intrinsically self-healable materials with high healing efficiencies and high tensile strengths. Intrinsic self-healing requires that fractured surfaces be bridged before healing. However, high tensile strength materials, often associated with higher T_g_ values, do not have adequate flow for this key step to occur. If the ambient temperature is below the T_g_, chain-end mobility will be too restricted for interchain diffusion and/or chain end recombination, and the efficiency of self-healing will be low [143]. Consequently, most examples of intrinsically self-healing materials are elastomers. The most recent 4th generation of self-healing materials is based on the combination of two or more intrinsically self-healing motifs (the 3rd generation is not pertinent to this review). This approach circumvents problems associated with balancing healing ability and strength [6]. Self-healing materials combining dual-non-covalent, dual-covalent, and a combination of non-covalent and covalent healing motifs have been reported.

Dual-responsive, fourth generation self-healing materials have been made by pairing disulfides with shape memory materials [14,32,144,145,146,147], as well as iminie [17,148,149] hydrogen [143,150,151,152,153,154,155,156,157,158], and metal-ligand bonds [159]. Self-healing in the presence of a stimulus, such as heat or UV radiation, is commonly reported but a greater emphasis has been placed on developing systems that can heal in ambient conditions [13]. If the T_g_ of the material is above room temperature, an added stimulus will be necessary for self-healing. This is part of the trade-off that is often required if strong materials are desired. Since disulfide-disulfide metathesis generally drives material repair, aryl disulfides, which are more reactive in metathesis reactions, have been popular in self-healing material design.

#### 3.2.2. Quantification of Self-Healing Behavior

Healing time and healing efficiency are commonly monitored via optical techniques. The size of a scratch or crack can be monitored over time, and the ratio of the initial crack to the crack width at a time can be used to calculate the self-healing efficiency.

Ultimate tensile strength (UTS) can be measured before and after self-healing to quantify self-healing efficiency using the method of Wool and O’Connor, which is expressed in equation (1), where x is a tensile property such as strength, elongation at break, or robustness [160].

(1) $$\mathrm{self}-\mathrm{healing}\;\mathrm{efficency}=\frac{x_{healed}}{x_{virgin}}\times 100\%$$

Comparing the self-healing efficiencies found from different studies, or finding using different methods in one study, can be problematic because many variables can influence the result. For example, the size of the initial cut and positioning of the cut pieces can influence self-healing time and efficiency measurements. Additionally, for tensile studies, changes in the thin film thickness and stretching rate can result in different tensile property values. Some studies also report the stress at which the material breaks when stretched. The UTS of a material is always less than or equal to the yield strength, which is the stress above which permanent deformation occurs. When considering the self-healing data in Table 2, these factors should be kept in mind.

#### 3.2.3. Disulfide-Based Self-Healing

The tensile strengths and strains at the break points of the pristine materials and parameters related to self-healing are included in Table 2. Those data demonstrate the variety of different disulfides that have been incorporated into self-healing polymer networks. They include aryl [13,14,17] and aliphatic [32,84,143,161] disulfides. More exotic disulfides, such as bis(2,2,6,6-tetramethylpiperidin-1-yl)disulfides (BiTEMPS) [162,163], and thiuram disulfide [164] have been incorporated into polymeric networks as well.

**Figure 33:**
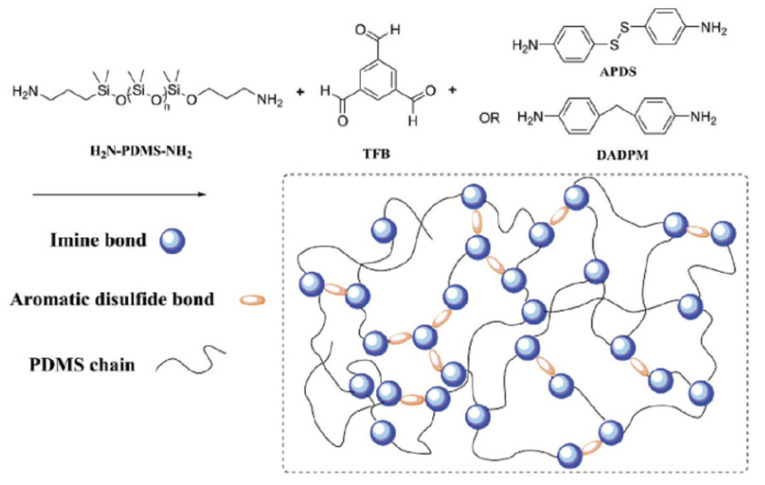
Dual imine- and disulfide-functionalized PDMS elastomers synthesized from aminopropyl poly(dimethyl siloxane), 1,3,5-triformyl benzene (TFB), and either 4-aminophenyl disulfide (APDS) or 4,4′-aminophenyl methane (DADPM). Reaction with APDS resulted in a dual-functionalized network, whereas reaction with DADPM resulted in a mono-functionalized imine network. Reprinted with permission from [17]. Copyright (2017) Wiley Periodicals, Inc.

Lv and coworkers [17] prepared dual-self-healing polydimethylsiloxane (PDMS) elastomers containing two types of dynamic covalent bonds, aromatic disulfide and imine linkages (Figure 33).

Imine bonds were incorporated into the PDMS network as temporary crosslinks, whereas disulfide bonds were incorporated as “sacrificial bonds” that could break and reorganize in response to mechanical stress. The elastomers were reprocessable and could self-heal completely within 4 h. Tensile testing for the dual-functionalized PDMS elastomers showed strain at break values of up to 2000% if stretched slowly. The strain at break of the dual-functionalized PDMS was higher than a control PDMS containing only imine groups. The disulfide bonds appear to function as sacrificial bonds during stretching and be responsible for the high elasticity. Cyclic tensile tests on the dual-functionalized elastomers revealed a pronounced hysteresis between subsequent stress loading and unloading cycles. However, when the material was left undisturbed for 2 days at room temperature, the initial stress loading/unloading curve was reestablished.

The self-healing ability of dual-functionalized PDMS was better than that of imine single-functionalized PDMS: 100% self-healing was attained in 4h for the dual-functionalized PDMS and 55% in 24h for the imine single-functionalized material. The dual-functionalized PDMS was reprocessed using cold-compression modeling, and the decrease in tensile strength was negligible after three repeat reprocessing steps. The dual-functionalized PDMS was degraded due to imine bond scission when the pH was lowered, or when an aldehyde or alkoxyamine was added. Although not examined, the effects of disulfide bond cleavage reagents would be interesting to examine, because this system is probably sensitive to stimulus-induced degradation and self-healing.

**Figure 34:**
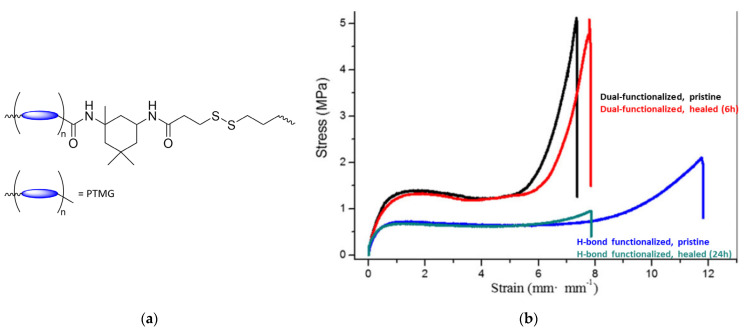
(**a**) Structure of dual-functionalized polyurethanes incorporating poly(tetramethylene ether) glycol (PTMG). (**b**) Tensile stress versus strain curves before and after self-healing for dual-functionalized PU and a control material with only hydrogen bonding Adapted with permission from [143]. Copyright (2017) Wiley Periodicals, Inc.

Jian and coworkers [143] examined dual-functionalized polyurethane (PU) that contained disulfide bonds with a strong hydrogen bonding motif for advanced self-healing applications (Figure 34a). Although the self-healing associated with the disulfides occurred over longer timescales than that associated with hydrogen bonding, it was key to full self-healing, as shown by comparison of the tensile curves of dual-functionalized and hydrogen bond mono-functionalized PUs (Figure 34b). However, hydrogen bonding interactions accounted for almost 50% of the early self-healing.

**Table 2:** Self-Healing Polymers Incorporating Disulfide Linkages.

Polymer Structure	Secondary Self- Healing Interaction	External Stimuli	Self-Healing Efficency ^1^	Time to Self-Heal ^2^	Tensile Strength ^3^ (MPa)	Strain at Break ^3^	Reference
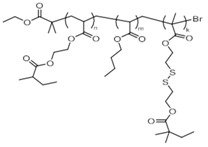 polyEGDA-polyBA star polymers	none	none	100%	Variable ^4^	n/a	n/a	[84]
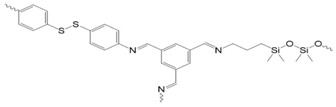 random PDMS-based co-polymer	imine bonding	none ^5^	95%	4 h	0.14	2200%	[16]
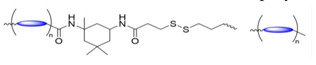 = PTMG	H-bonding	60 °C	100%	6 h	5.01	n/a	[143]
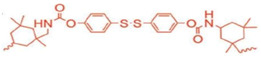 IP-SS: co-polymerized with PTMG	none	25 °C	77–100%	2 h	6.8	920%	[12]
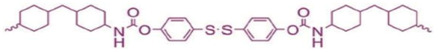 4,4’-methylenebis(phenyl isocyanate) based copolymer of PTMG (HM-SS)	none	25 °C	70–89%	2 h	4.5	490%	[12]
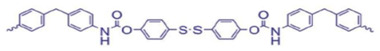 M-SS: co-polymerized with PTMG	none	25 °C	0–4%	2 h	30.4	940%	[12]
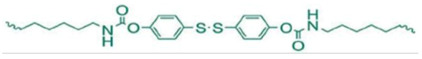 H-SS: co-polymerized with PTMG	none	25 °C	4–30%	2 h	9.5	750%	[12]
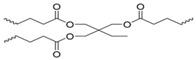 co-polymerized with 2-ethylhexyl methacrylate via disulfide linkages	none	none	100% ^7^	3–30 min.	n/a	n/a	[161]
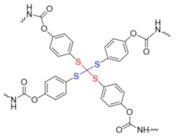 co-polymerized with PTMG	shape memory	100 °C (microwave)	74–91% ^6^	10 min.	22.9–31.9 ^6^	850–1160% ^6^	[13]
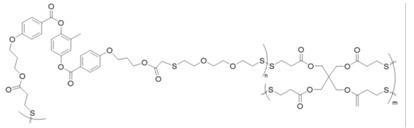 disulfide-liquid crystal elastomer	shape memory	$\frac{180\;{}^{\circ}C}{\mathrm{RT},\;\mathrm{UV}\;\mathrm{light}}$	$\frac{20\%}{24\%}$	$\frac{3\;h}{3\;h}$	0.015–0.12%	350%	[32]
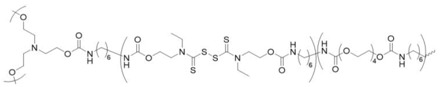 thiuram disulfide crosslinked polytetra(ethylene glycol)	none	table-top lamp	87–97%	1 min.	4.2	200%	[163]
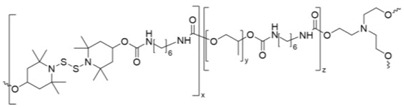 PU incorporating BiTEMPS	none	100 °C	86–93%	24 h	0.14	440%	[162]
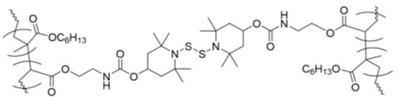 poly(hexyl methacrylate) incorporating BiTEMPS	none	120 °C, 70 kPa	85–92%	24 h	7.6	280	[164]

^1^ Self-healing efficencies are reported as ranges in some cases because different self-healing method. ^2^ For healing at room temperature (RT), if more than one temperature tested. ^3^ For.pristine materials. ^4^ Self-healing time varried based upon the width and depth of a cut. ^5^ For cutting and reprocessing studies, pressure was 10 MPa; atmospheric pressure for scratch tests. ^6^ Depended on disulfide content. ^7^ Healing based on the change in the cut width.

The results of the groups of Lv [17] and Jian [143] suggest that disulfide bonds are more effective as self-healers than imine or hydrogen bonds. However, disulfide-based self-healing appears to occur over a longer time scale. Pairing disulfide bonds with the faster self-healing functionalities may improve overall self-healing and material longevity by lowering the risk of permanent deformation when rapid stress is applied.

**Figure 35:**
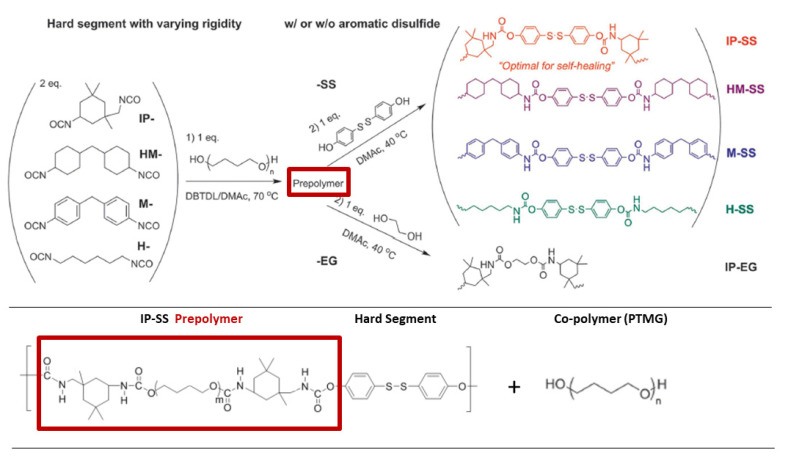
Synthesis of diphenyl disulfide “hard segment” prepolymers and subsequent copolymerization with PTMG. Adapted with permission from [13]. Copyright (2017) Wiley Periodicals, Inc.

Kim and co-workers [13] examined how structure-property relationships influenced self-healing in thermoplastic polyurethanes (TPUs) containing aryl disulfide groups with variable molecular packing and rigidities. Thus, ‘hard’ *para*-substituted diaryl disulfide prepolymers were copolymerized with ‘soft’ poly(tetramethylene ether) glycol segments (PTMG) (Figure 35). Comparison of the results from the resultant copolymers suggests that the packing ability of the hard segment, which is determined by the R groups flanking the aryl disulfide, strongly influences self-healing. TPUs formed with an alicyclic isophorone diisocyanate-based hard segment (IP-SS) self-healed most efficiently (Table 2). The tensile strength of IP-SS was 6.8 MPa, which is nearly one order of magnitude higher than that reported for crosslinked PUs (0.8 MPa) [150]. This is one of the highest strengths reported among materials that can self-heal at room temperature.

Kim and coworkers propose that the combination of the rigid diaryl disulfide and the alicyclic flanking groups provides sufficient chain mobility for efficient healing without weakening the material to an appreciable extent. By comparison, TPUs formed with symmetric alicyclic 4,4′-methylenebis(phenyl isocyanate) (M-SS) and linear aliphatic hexamethylene diisocyanate hard segments (H-SS), exhibited little self-healing ability. The higher tensile strengths of M-SS and H-SS (Table 2) than that of IP-SS suggests that the rigidity of M-SS and the high packing ability of H-SS hinder self-healing. Although both M-SS and H-SS exhibited no self-healing in scratch tests, some recovery of tensile properties was observed (especially for H-SS, see values in Table 2) when the materials were cut and subjected to pressure.

**Figure 36:**
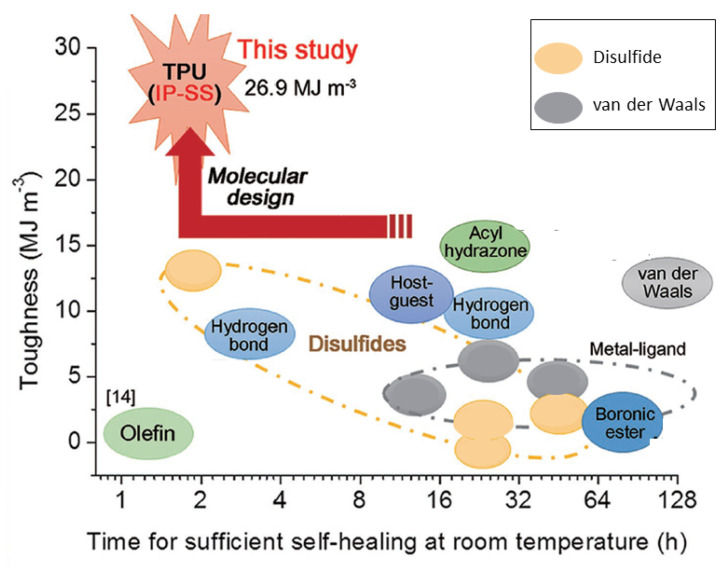
Ashby plot of self-healing time at room temperature versus toughness. Adapted with permission from [13]. Copyright (2017) Wiley Periodicals, Inc.

An Ashby plot of self-healing time at room temperature versus toughness (Figure 36) highlights the superior performance of IP-SS compared to other intrinsically self-healing materials. As shown in Table 2, self-healing materials with comparable or higher tensile strengths have been reported, but they require heat, pressure, and/or light for comparably efficient self-healing to occur [14,143,163].

## 4. Conclusions

Thiomers and disulfide-containing polymers have been studied for a wide variety of applications, due to their stimulus-responsive reactions. Many applications for these materials have been proposed and realized. Since disulfide-based drug delivery systems were first approved for human use nearly 20 years ago, research on them and an expansion of their forms has led to a wide range of sustainable materials that exhibit self-healing and controllable biodegradation. Future research in the field of thiomer and disulfide-containing polymers as in drug delivery agents and self-healing materials appears to be ongoing at an ever-increasing pace. We expect that future research will explore the potential of multi stimulus-responsive materials that include other functionalities moreover disulfides and thiols.
